# Flp/*FRT*-mediated disruption of *ptex150* and *exp2* in *Plasmodium falciparum* sporozoites inhibits liver-stage development

**DOI:** 10.1073/pnas.2403442121

**Published:** 2024-07-05

**Authors:** Robyn McConville, Jelte M. M. Krol, Ryan W. J. Steel, Matthew T. O’Neill, Bethany K. Davey, Anthony N. Hodder, Thomas Nebl, Alan F. Cowman, Norman Kneteman, Justin A. Boddey

**Affiliations:** ^a^Division of Infectious Diseases & Immune Defence, The Walter and Eliza Hall Institute of Medical Research, Parkville, VIC 3052, Australia; ^b^Department of Medical Biology, University of Melbourne, Melbourne, VIC 3010, Australia; ^c^Departments of Surgery, University of Alberta, Edmonton, AB T6G 2E1, Canada

**Keywords:** malaria, protein export, translocon, hepatocyte, humanized mice

## Abstract

*Plasmodium falciparum* causes severe malaria and assembles a protein translocon (PTEX) complex at the parasitophorous vacuole membrane (PVM) of infected erythrocytes, through which several hundred proteins are exported to facilitate growth. The preceding liver stage of infection involves growth in a hepatocyte-derived PVM; however, the importance of protein export during *P. falciparum* liver infection remains unexplored. Here, we use the FlpL/*FRT* system to conditionally excise genes in *P. falciparum* sporozoites for functional liver-stage studies. Disruption of PTEX members *ptex150* and *exp2* did not affect sporozoite development in mosquitoes or infectivity for hepatocytes but attenuated liver-stage growth in humanized mice. While PTEX150 deficiency reduced fitness on day 6 postinfection by 40%, EXP2 deficiency caused 100% loss of liver parasites, demonstrating that PTEX components are required for growth in hepatocytes to differing degrees. To characterize PTEX loss-of-function mutations, we localized four liver-stage *Plasmodium* export element (PEXEL) proteins. *P. falciparum* liver specific protein 2 (LISP2), liver-stage antigen 3 (LSA3), circumsporozoite protein (CSP), and a *Plasmodium berghei* LISP2 reporter all localized to the periphery of *P. falciparum* liver stages but were not exported beyond the PVM. Expression of LISP2 and CSP but not LSA3 was reduced in *ptex150-FRT* and *exp2-FRT* liver stages, suggesting that expression of some PEXEL proteins is affected directly or indirectly by PTEX disruption. These results show that PTEX150 and EXP2 are important for *P. falciparum* development in hepatocytes and emphasize the emerging complexity of PEXEL protein trafficking.

## Significance

*Plasmodium falciparum* causes the most lethal malaria and exports several hundred proteins through the PTEX translocon into the erythrocyte. While malaria parasites infect hepatocytes before erythrocytes, the importance of protein export during *P. falciparum* liver infection remains unexplored. We adapted the Flp/*FRT* system to conditionally disrupt genes in *P. falciparum* sporozoites for functional studies. We show that translocon members PTEX150 and EXP2 are expressed throughout the liver stage and are essential for *P. falciparum* growth in hepatocytes. Analysis of liver stage PEXEL proteins shows they localize to the parasite periphery. A subset of P. falciparum pre-erythrocytic effectors may be trafficked into the host cell, however, targeting to the parasite-host interface may facilitate host cell subversion whilst limiting antigen presentation.

*Plasmodium* species have a complex lifecycle involving transmission between mosquitoes and humans. Sporozoites deposited into the skin during mosquito feeding infect the liver, where they develop as exoerythrocytic forms (EEFs) within hepatocytes. Subsequently, EEFs egress in merosomes and infect erythrocytes, leading to the symptoms of malaria. In 2022, there were 249 million malaria cases and 608,000 deaths (1).

A feature of *Plasmodium falciparum* infection is remodeling of the infected erythrocyte by exported proteins. Such modifications allow parasites to sequester in the microvasculature, evade immune responses, and replicate (2). Export involves protein transport from the endoplasmic reticulum (ER) to the parasitophorous vacuole (PV) and across the PV membrane (PVM). The molecular mechanisms of protein export have been studied predominantly in *P. falciparum* asexual blood stages (BS). One class of exported proteins contains the *Plasmodium* export element (PEXEL) (3, 4), which consists of the motif RxLxE/Q/D that is cleaved in the ER (5, 6) by plasmepsin V (7, 8). This protease is located in an ER complex that selects proteins for export (9). Over 350 *P. falciparum* proteins contain a PEXEL sequence (10–12). Once cleaved by plasmepsin V, some PEXEL proteins are bound by chaperones, including HSP101 (8, 13), and transported to the PV (6) for translocation across the PVM (14). This step involves the *Plasmodium* translocon of exported proteins (PTEX), which is composed of EXP2, PTEX150, HSP101, PTEX88, and TRX2 (15–18) and three associated proteins PV1, Pf113, and HSP70-x (19, 20). EXP2 also functions as a nutrient transporter (21), which requires EXP1 (22). PEXEL-negative exported proteins (PNEPs) (23, 24) also traffic across the PVM through this translocon (16, 17). PTEX, plasmepsin V, PEXEL, and PNEP proteins are also expressed and essential in *P. falciparum* sexual stages (17, 25–27) and are attractive targets for drug development (2, 18, 28).

As the first stage of mammalian infection, the *Plasmodium* liver stage offers unique opportunities for intervention (29). Liver stages infect and alter the hepatocyte in several ways to facilitate their survival (30, 31). To date, three liver-stage proteins have been shown to be exported to the hepatocyte cytosol: circumsporozoite protein (CSP) (32), liver specific protein 2 (LISP2) (33), and sporozoite and liver-stage tryptophan-rich protein (SLTRiP) (34); interestingly, SLTRiP is annotated as a pseudogene in *Plasmodium berghei*. Cleavage of LISP2 occurs in *P. berghei* EEFs, within the PEXEL following the conserved leucine residue, which was detectable in merosomes (35), suggesting that plasmepsin V cleavage of the PEXEL of exported proteins occurs during hepatocyte infection, but this remains to be formally proven. Several chimeric PEXEL fusions are also exported by *P. berghei* EEFs into the hepatocyte (36–38). Interestingly, numerous PEXEL proteins localize at the PV/PVM rather than the host cell (35, 39–42). Several recently characterized *P. falciparum* PEXEL proteins also localize at the PV/PVM (43–45). While this peripheral localization could be interpreted as non-exported, it is possible that the protein binds to the cytoplasmic face of the PVM via a peripheral protein-protein interaction following export and so biochemical assessment of membrane binding and topology is needed in order to be conclusive. Localization in the PVM also constitutes export if a protein domain is exposed to the host cell.

The function of PTEX in liver stages remains to be proven, but it is speculated to be involved in protein export. There is evidence that PTEX component EXP2 is expressed and important for *P. berghei* liver-stage infection (42, 46). *P. falciparum* is the pathogen causing the most severe form of malaria in humans and protein export during liver infection may hold promise as a target for preventing malaria and for vaccination if exported proteins are presented to the immune system. However, protein export has not been functionally explored at the liver stage of the human malaria parasite.

*P. falciparum* EEFs express PTEX components PTEX150 and EXP2 as well as the PEXEL proteins CSP and Liver Stage Associated Protein 2 at the parasite periphery during the late liver stage, likely at the PVM (47, 48). Whether PTEX is necessary for *P. falciparum* growth and/or protein export during hepatocyte infection remains unknown and is the basis of the current study.

Here, we show that PTEX members are expressed in early, mid, and late liver stages and used the FlpL/*FRT* conditional system (49, 50) to disrupt *ptex150* and *exp2* in *P. falciparum* sporozoites. PTEX loss-of-function mutants were inhibited for growth in livers of humanized mice and showed reduced expression of the PEXEL proteins LISP2 and CSP. Four liver-stage PEXEL proteins were localized to the EEF periphery rather than in the host cell, precluding functional characterization of PTEX translocation activity. Therefore, *P. falciparum* requires PTEX150 and EXP2 for intracellular growth across the lifecycle and targets PEXEL proteins to the periphery as well as the host cell.

## Results

### *P. falciparum* Expresses PTEX150 and EXP2 at the Early, Mid, and Late Liver Stages.

**Fig. 1.**
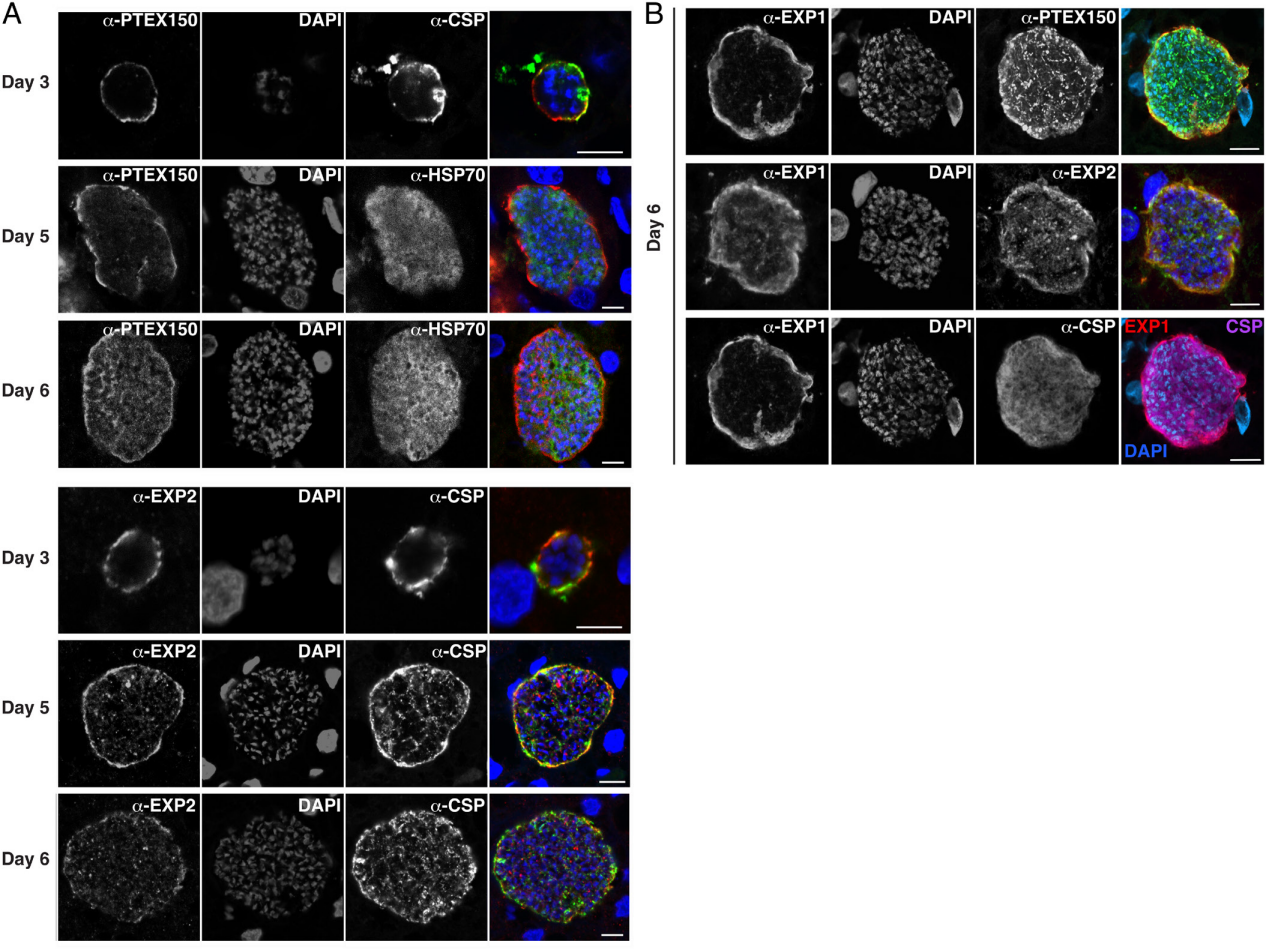
Expression of PTEX150 and EXP2 during *P. falciparum* liver infection in humanized mice. Sections were co-stained with (*A*) PTEX150, EXP2, CSP (periphery) or HSP70 (cytoplasm); and (*B*) PTEX150, EXP2, EXP1 (PVM) or CSP (periphery). Scale bar, 10 um.

To understand when PTEX is expressed by *P. falciparum* EEFs, we performed immunofluorescence microscopy assays (IFA) on sections of chimeric human livers from humanized mice at different timepoints using antibodies specific to PTEX150, EXP2, and EEF markers EXP1 (PVM), HSP70 (cytoplasm), and CSP (periphery). It has been shown previously that *P. falciparum* EEFs express PTEX150 and EXP2 on days 5 and 7 postinfection in humanized mice (47). During infection of erythrocytes, PTEX is assembled very early, following invasion, ensuring export occurs soon after entry into the cell (15). During liver infection, we observed PTEX150 and EXP2 expression inside the EEF and at the periphery in early (day 3), mid (day 5) and late (day 6) liver stages (Fig. 1*A*). The peripheral PTEX150 and EXP2 signals overlapped with the EXP1-labeled PVM, suggesting that these proteins were secreted from the parasite to the PV and/or PVM, while CSP was also observed colocalizing, at least partially, with PTEX150, EXP2, and EXP1 (Fig. 1*B*). The internal localization of PTEX150 and EXP2 observed late in development could be protein synthesis and trafficking to dense granules of newly formed merozoites ready for erythrocyte invasion. Liver-stage expression on each of the days tested suggested that PTEX components are present earlier than previously appreciated and may have an important function beginning soon after infection of the hepatocyte. As PTEX is essential for *P. falciparum* blood-stage growth (15–17), we next sought to establish a conditional mutagenesis system to enable functional characterization specifically in EEFs.

### Establishing the FlpL/*FRT* System in *P. falciparum* Sporozoites.

To characterize PTEX function in EEFs, we adapted the FlpL/*FRT* system (49) to *P. falciparum* mosquito stages. The gene encoding the thermolabile FlpL recombinase from *Saccharomyces cerevisiae* was integrated into the *P. falciparum* NF54 *rh3* pseudogene (PF3D7_1252400) by double cross-over homologous recombination using clustered regularly interspaced short palindromic repeat’ (CRISPR)/“CRISPR-associated protein 9” (Cas9) gene editing (*SI Appendix*, Fig. S1*A*). FlpL expression was regulated by the *P. falciparum trap* 5′ and 3′ elements, such that expression was minimal in asexual and sexual BS when PTEX is essential but up-regulated at sporogony, following parasite transmission to mosquitoes. Flp has been used previously in *P. berghei* liver stages (50) and *P. falciparum* BS (51, 52). FlpL was selected due to efficiency at 23 to 30 °C, temperatures used to maintain *P. falciparum*–infected mosquitoes. Clonal NF54 *trap*-FlpL parasites were generated by transfection and validated by Southern blot (*SI Appendix*, Fig. S1*B*). As a control, *P. falciparum* NF54 was transfected with a similar plasmid containing a noncoding exogenous DNA fragment in place of the *trap*-FlpL, thereby retaining PTEX expression (called Control 1; *SI Appendix*, Fig. S2). The noncoding DNA fragment allowed differentiation of the control genome from both FlpL/*ptex*-*FRT* genomes.

**Fig. 2.**
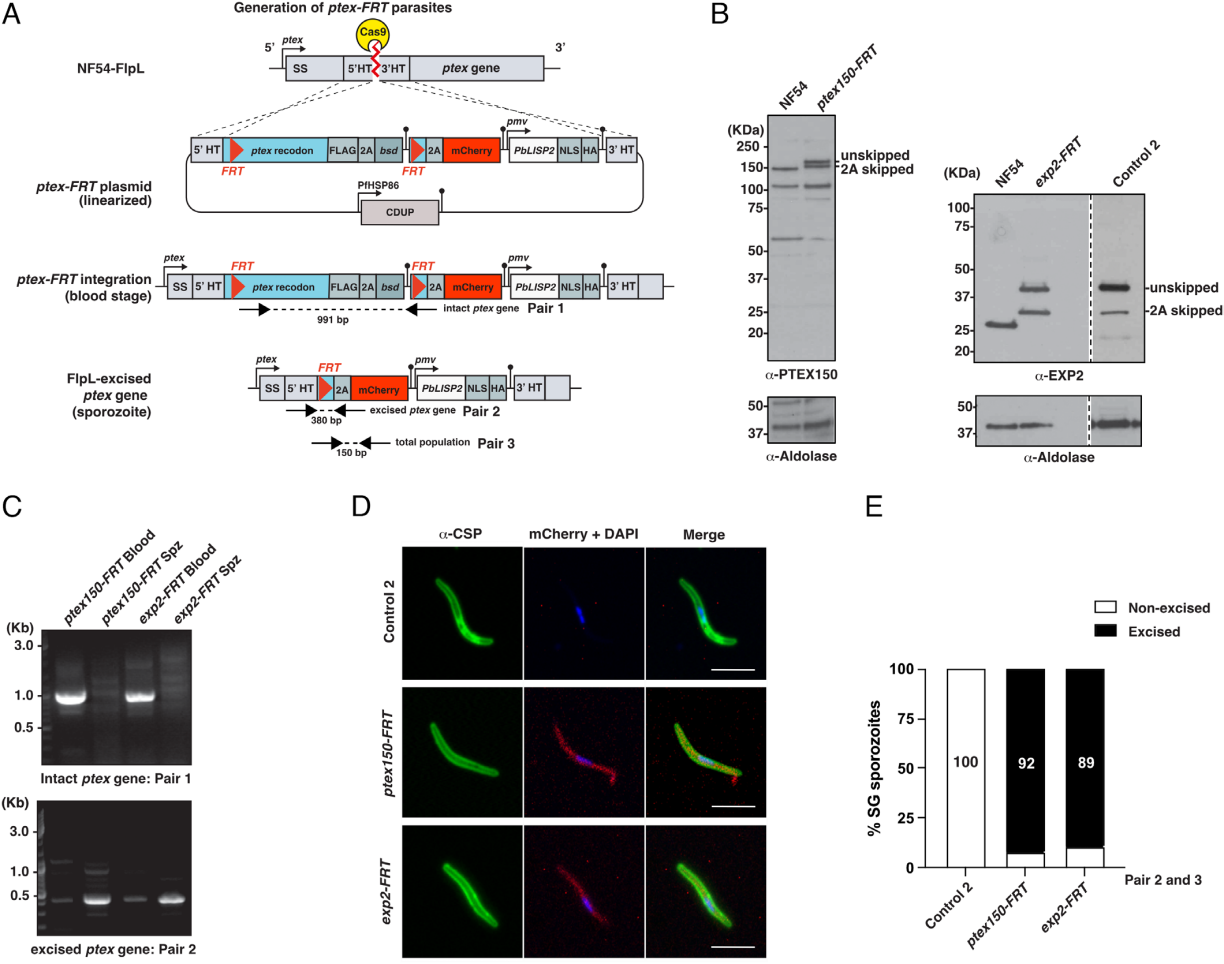
Conditional disruption of *ptex150* and *exp2* genes in *P. falciparum* sporozoites. (*A*) Strategy to integrate each *ptex-FRT* plasmid (*ptex150-FRT* or *exp2-FRT*) into NF54 *trap*-FlpL parasites by double cross-over homologous recombination. Constructs contained blasticidin deaminase (*bsd*) for positive selection and cytosine deaminase (CDUP) for negative selection. HT, homology target. (*B*) Immunoblot confirming integration of *ptex150-FRT* and *exp2-FRT* plasmids into the endogenous locus in NF54 *trap*-FlpL parasites. Aldolase loading control is ~40 kDa. (*C*) PCR analyses confirming integration of *ptex150-FRT* and *exp2-FRT* plasmids in blood-stage parasites (Blood) and excision of both loci in sporozoites (Spz) after mosquito transmission. (*D*) Immunofluorescence microscopy shows excision of *ptex150-FRT* and *exp2-FRT* in sporozoites, determined by mCherry expression. (Scale bar, 5 mm.) (*E*) Efficiency of *trap*-FlpL excision of *ptex150-FRT* and *exp2-FRT* quantified by qRT-PCR with primer pairs 2 and 3 (refer to panel *A*).

To enable genetic disruption of *ptex15*0 and *exp2* in sporozoites, NF54 *trap*-FlpL or Control 1 parasite lines were further genetically modified by transfection with a construct that replaced each *ptex* gene with DNA consisting of three components: a codon-optimized version of each *ptex* gene flanked by *FRT* sites within introns for conditional excision, an mCherry reporter to confirm *FRT* recombination by FlpL in sporozoites, and a chimeric LISP2 PEXEL reporter from *P. berghei* (PbLISP2-NLS-HA) to assess protein export in hepatocytes (Fig. 2*A* and *SI Appendix*, Fig. S3). Replacement of the endogenous *ptex150* and *exp2* genes with the codon-optimized *ptex-FRT* alleles was performed by double cross-over homologous recombination using CRISPR/Cas9. The *ptex150-FRT* and *exp2-FRT* constructs were transfected into NF54 *trap*-FlpL parasites (Fig. 2*A*) or Control 1 parasites lacking *trap*-FlpL (*SI Appendix*, Fig. S3), and integration confirmed by immunoblotting with anti-PTEX150 and anti-EXP2 antibodies that detected an expected mass increase from incorporation of a C-terminal FLAG tag (Fig. 2*B*, see “2A skipped” bands). Separation of the polypeptides by 2A skip was incomplete, as evidenced by a larger protein species representing PTEX150-FLAG or EXP2-FLAG proteins fused to BSD (Fig. 2*B*, see “unskipped” bands). However, asexual blood-stage growth and gametocytogenesis occurred without apparent issue, allowing transmission of all parasite lines to mosquitoes. Successful integration of the *exp2-FRT* construct into Control 1 parasites lacking *trap*-FlpL resulted in Control 2 parasites used in this study (Fig. 2*B*). Genotyping by PCR confirmed that the integrated *ptex150-FRT* and *exp2-FRT* loci were intact in asexual BS as expected but excised in sporozoites that contained *trap*-FlpL (Fig. 2*C*). Excision of each *ptex* locus was confirmed by mCherry expression by IFA (Fig. 2*D*) and quantitative reverse-transcriptase PCR (qRT-PCR) confirmed that FlpL was efficient at the *ptex150-FRT* and *exp2-FRT* loci in sporozoites (Fig. 2*E*). Altogether, these results demonstrate that the FlpL/*FRT* system was efficient at conditionally deleting *ptex* genes in *P. falciparum* sporozoites.

### *ptex150-FRT* and *exp2-FRT* Transmission Stages Retain Infectivity.

**Fig. 3.**
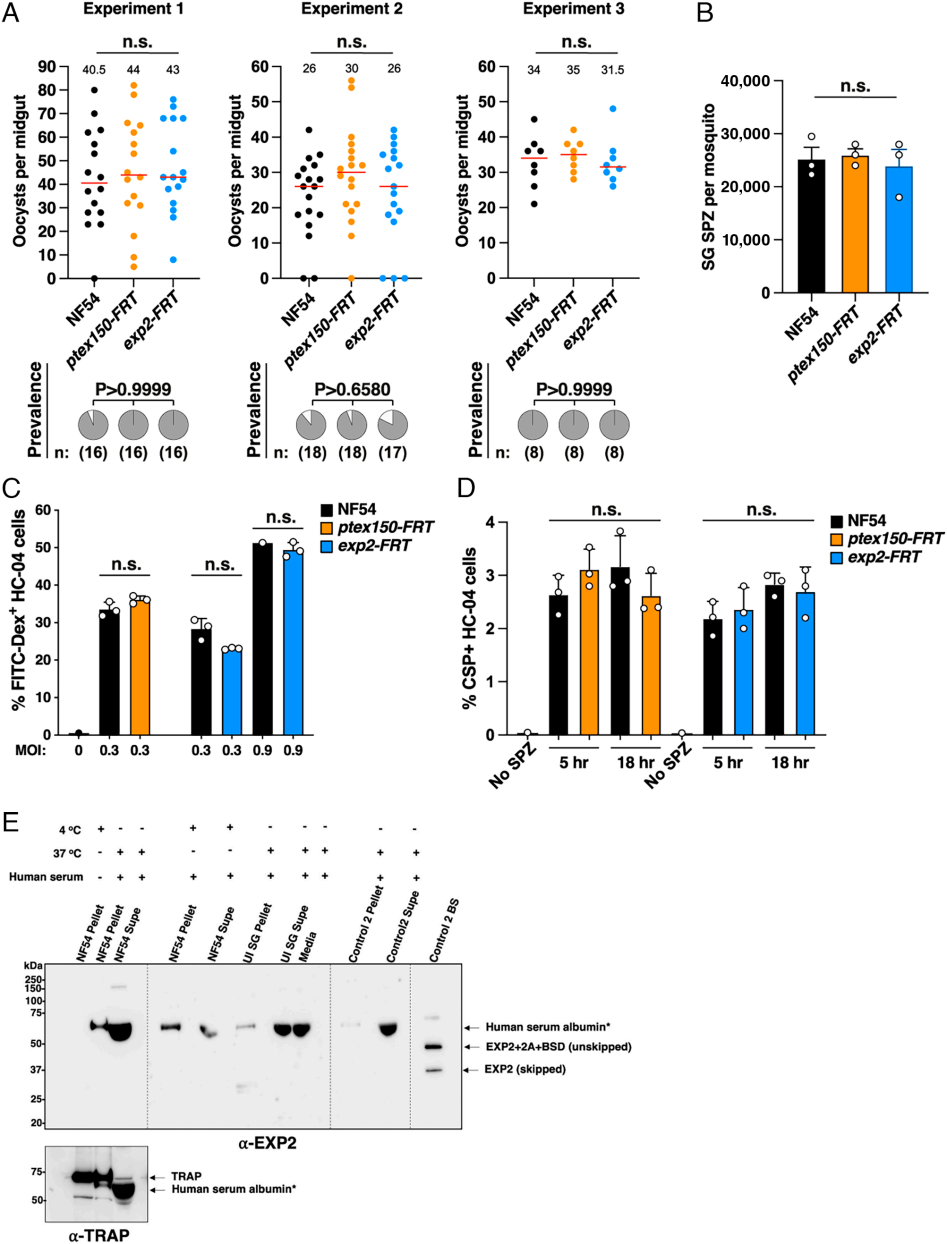
Development and infectivity of *P. falciparum* PTEX mutant sporozoites. (*A*) Mosquito oocyst intensity (*Top*) and infection prevalence (*Bottom*) following standard membrane feeding assays. The mean oocyst number is indicated by a red bar and small number. The mosquito sample size (n:) is shown. Oocysts were compared using the Kruskal–Wallis one-tailed test with Dunn’s correction, and prevalence was compared using the chi-square test (Fisher’s exact test). Data are from three independent experiments. *P* values are indicated; n.s., not significant. (*B*) Salivary gland sporozoite (SG SPZ) counts per mosquito. Data are from three independent experiments. (*C*) HC-04 cell traversal by *P. falciparum* sporozoites at multiplicity of infection (MOI) 0.3 and 0.9, measured by FITC-Dextran uptake. (*D*) HC-04 invasion by *P. falciparum* sporozoites measured after incubation for 5 and 18 h, using CSP-positive antibody staining of fixed permeabilized cells. Data in panels (*B*–*D*) are mean ± SEM from n = 3 experiments analyzed by one-way ANOVA (Kruskal–Wallis test). n.s., not significant. (*E*) Immunoblot of *P. falciparum* sporozoites and Control 2 BS for EXP2 expression. The overexposure is to demonstrate the absence of EXP2. Sporozoites (1 million per condition) were incubated under the designated conditions and probed with anti-EXP2 and anti-TRAP control antibodies (*Left* blot). *Cross-reactivity of human serum albumin in the media in numerous lanes is indicated at circa 65 kDa. “EXP2+2A + BSD (unskipped)” is a size control of unskipped EXP2 fused to 2A and Blasticidin S Deaminase (BSD) if EXP2 was expressed in Control 2 sporozoites. “EXP2 (skipped)” is a size control of 2A skipped EXP2 if it was expressed in NF54 or Control 2 sporozoites. Uninfected salivary glands (UI SG) were treated as above as a negative control. Lysates from Control 2 BS were used as a positive control for EXP2 expression.

To study the function of PTEX in EEFs, we differentiated asexual parasites to stage V gametocytes and transmitted them to *Anopheles stephensi* mosquitoes. Quantification of mosquito infections revealed no defect in the formation of midgut oocysts or infection prevalence between *ptex* mutants and controls (Fig. 3*A*). Similarly, no defect was detected in the number of salivary gland sporozoites produced by all parasite lines (Fig. 3*B*). We next investigated the ability of *ptex* mutant sporozoites dissected from mosquito salivary glands to traverse human HC-04 hepatocytes in vitro and did not detect any differences in the rate of cell traversal between parasite lines (Fig. 3*C*), suggesting that they were similarly motile and infectious. Finally, we assessed whether *ptex* mutant sporozoites could invade HC-04 hepatocytes (53) and again observed no defect in the number of invaded cells between parasite lines at either 5- or 18-h postinfection (Fig. 3*D*). Therefore, *ptex150-FRT* and *exp2-FRT* transmission stages retained apparently similar infectivity to Controls.

The results for sporozoite invasion into HC-04 cells contrasted with a prior study showing that excision of the *exp2* 3′UTR in *P. berghei* sporozoites impaired invasion of HepG2 cells (54). This discrepancy could be due to differences in the technical/genetic/methodological approaches, or the parasite species between studies. We therefore investigated whether *P. falciparum* sporozoites express EXP2. Unlike in *P. berghei* sporozoites (54), immunoblot of one million *P. falciparum* NF54 sporozoites failed to detect EXP2 expression using antibodies raised previously to this protein (15), including after incubation of sporozoites with fetal calf serum at 37 °C to activate them as per the *P. berghei* study, whereas expression was detected by the antibody in asexual BS as expected (Fig. 3*E*). The lack of EXP2 expression in *P. falciparum* salivary gland sporozoites was unexpected given the previous results in *P. berghei* (54) and indicates a species-specific difference. Analysis of proteomics data for evidence of EXP2 expression in sporozoites did not detect EXP2 peptides from *P. falciparum, P. vivax*, *Plasmodium yoelii,* and *P. berghei* (55–59). Altogether, this suggests that *P. falciparum* NF54 likely does not express EXP2 in salivary gland sporozoites under the conditions tested, demonstrating why the sporozoites were not defective for HC-04 invasion.

### PTEX150 and EXP2 Are Required for *P. falciparum* Liver-Stage Development.

**Fig. 4.**
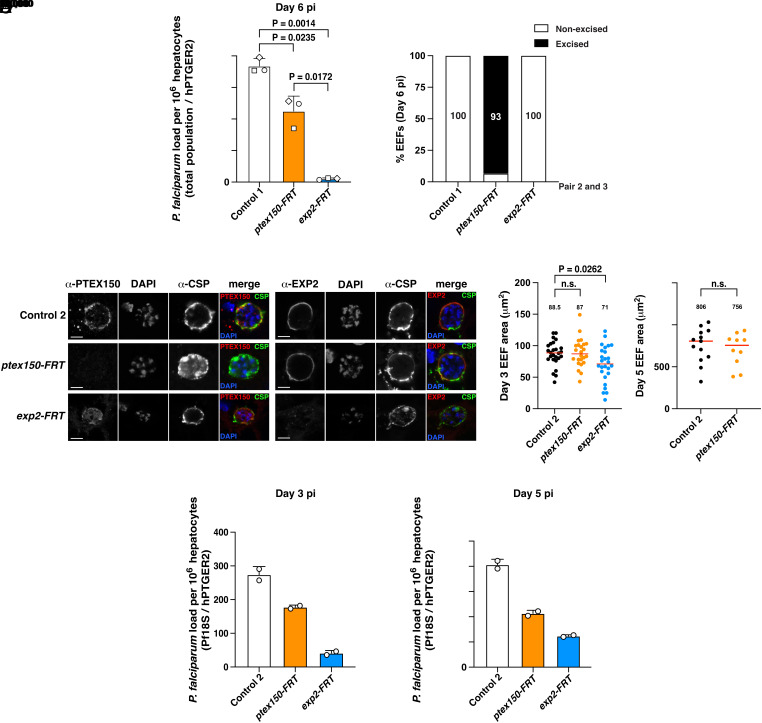
PTEX150 and EXP2 are required for *P. falciparum* liver-stage development. (*A*) Quantification of parasite liver load coinfected into mice with chimeric human livers 6 d postinfection. Three mice received equal numbers of Control 1, *ptex150-FRT,* and *exp2-FRT* sporozoites in the same i.v. injection. Control 1 was used due to its unique genotype relative to *ptex-FRT* lines, allowing quantification of each parasite line in the coinfected mice. Each mouse is indicated by a different shape. Parasite liver load determined by qRT-PCR, analyzed by one-way ANOVA (Kruskal–Wallis test). (*B*) Genotyping of EEFs on day 6 from 3 coinfected mice from panel *A* shows excision of *ptex150-FRT* and *exp2-FRT* loci quantified by qRT-PCR using primer pairs 2 and 3. (*C*) Immunofluorescence microscopy of liver sections from individually infected humanized mice on day 3 postinfection. Mice were infected separately with Control 2, *ptex150-FRT,* or *exp2-FRT* sporozoites. (Scale bar, 5 mm.) (*D*) Area of *P. falciparum* EEFs on day 3 (*Left*) or day 5 (*Right*) postinfection. Data are median, indicated with a number and a red bar within each condition, from n = 23 to 26 (day 3) or n = 10 to 13 (day 5) individual EEFs analyzed by Mann–Whitney *t* test. *P* values are shown; n.s., not significant. (*E*) Quantification of parasite liver load in each humanized mice that were individually infected with Control 2, *ptex150-FRT,* or *exp2-FRT* sporozoites in separate injections on day 3 (*Left*) and day 5 (*Right*) postinfection by qRT-PCR. Shown are n = 2 technical qRT-PCR replicates as mean ± SEM from one mouse per condition (three mice for day 3, three mice for day 5; six mice total) Values were normalized to a series of pretested DNA standards.

To investigate whether PTEX150 or EXP2 have a role in *P. falciparum* liver-stage development, we coinfected three humanized mice containing chimeric human livers with equal numbers of Control 1, *ptex150-FRT,* and *exp2-FRT* sporozoites in a single intravenous injection per mouse. Control 1 parasites were used because they contained a unique exogenous DNA insert (*SI Appendix*, Fig. S2), thereby allowing specific molecular quantification of each parasite line from the same mouse liver samples by qPCR. Livers were isolated from humanized mice on day 6 postinfection, representing the late liver stage, and parasite liver load was quantified by qPCR. A key advantage of this coinfection model (60, 61) is that it allowed direct comparisons of within-mouse fitness of each parasite line to each other. The parasite liver loads for *ptex150-FRT* and *exp2-FRT* were consistently reduced compared to Control 1 parasites, by approximately 40% (*P* = 0.0235) and 97% (*P* = 0.0014), respectively (Fig. 4*A*). Further, the liver load of *exp2-FRT* was approximately 95% lower than *ptex150-FRT* (*P* = 0.0172). Genotyping revealed that 93% of *ptex150-FRT* EEFs on day 6 had excised *ptex150,* indicating this population survived without the gene, while the remainder had the intact *ptex150* gene presumably due to lack of FlpL excision during sporogony (Fig. 4*B*). Conversely, all *exp2-FRT* EEFs on day 6 had the intact *exp2* gene, suggesting that parasites that excised *exp2* had all died and the remainder originated from sporozoites with a nonexcised *exp2* locus that underwent positive selection in humanized mouse livers (Fig. 4*B*). Therefore, *exp2* is critical for EEF development while *ptex150* is important for parasite fitness but some EEFs could survive without this gene, demonstrating that EEFs require PTEX members to differing degrees.

To perform microscopy of each parasite line during liver-stage development, we infected six humanized mice with one parasite line per mouse and subsequently isolated the livers for analysis at two time points: days 3 and 5 postinfection, to determine whether PTEX was important prior to day 6. We employed Control 2 in these experiments as they contained the *exp2-FRT* locus but lacked FlpL, thus retaining EXP2 expression.

IFAs of liver sections on day 3 postinfection showed that EXP2 localization at the periphery was unaffected by deletion of PTEX150 and vice versa (Fig. 4*C*). As PTEX150 and EXP2 partly colocalized with CSP and EXP1 (Fig. 1), we conclude that these proteins were localizing at the PVM (Fig. 4*C*). This indicates that each PTEX component as well as CSP was trafficked to the parasite periphery in the absence of the other PTEX component.

IFAs also revealed that *exp2-FRT* EEFs were smaller than Control 2 parasites on day 3 (Fig. 4*D*). Such mutant EEFs were difficult to find in liver sections on day 5, consistent with a strong developmental defect. The challenge of identifying *exp2-FRT* EEFs on day 5 precluded further comparative microscopy analyses at this timepoint. *ptex150-FRT* EEFs were similar in size to Control 2 parasites on day 3 and, while challenging to identify, were of a similar size to controls on day 5 (Fig. 4*D*).

To quantify parasite development in this second cohort of mice, we conducted qPCR analysis of livers. The parasite liver load for *ptex150-FRT* and *exp2-FRT* was reduced on day 3 and 5 compared to Control 2 (Fig. 4*E*). This suggested that some *ptex150-FRT* and *exp2-FRT* parasites had died on or before day 3 and were eliminated from the liver as they could not survive. The use of one mouse per time point did not permit statistical analyses; however, the results from these experiments and the coinfected mice on day 6 postinfection (Fig. 4*A*) altogether suggest that *ptex150* and *exp2* are important for *P. falciparum* liver-stage growth in humanized mice, including in the early stage of infection, with a reduction in parasite liver load and size of *exp2-FRT* EEFs evident by day 3 postinfection. The decrease in *ptex150-FRT* liver load despite the apparently normal size of the remaining EEFs suggests that PTEX150 was also required for normal parasite survival in the host liver.

### Antibodies to LISP2 Localize to the Periphery of *P. falciparum* EEFs.

**Fig. 5.**
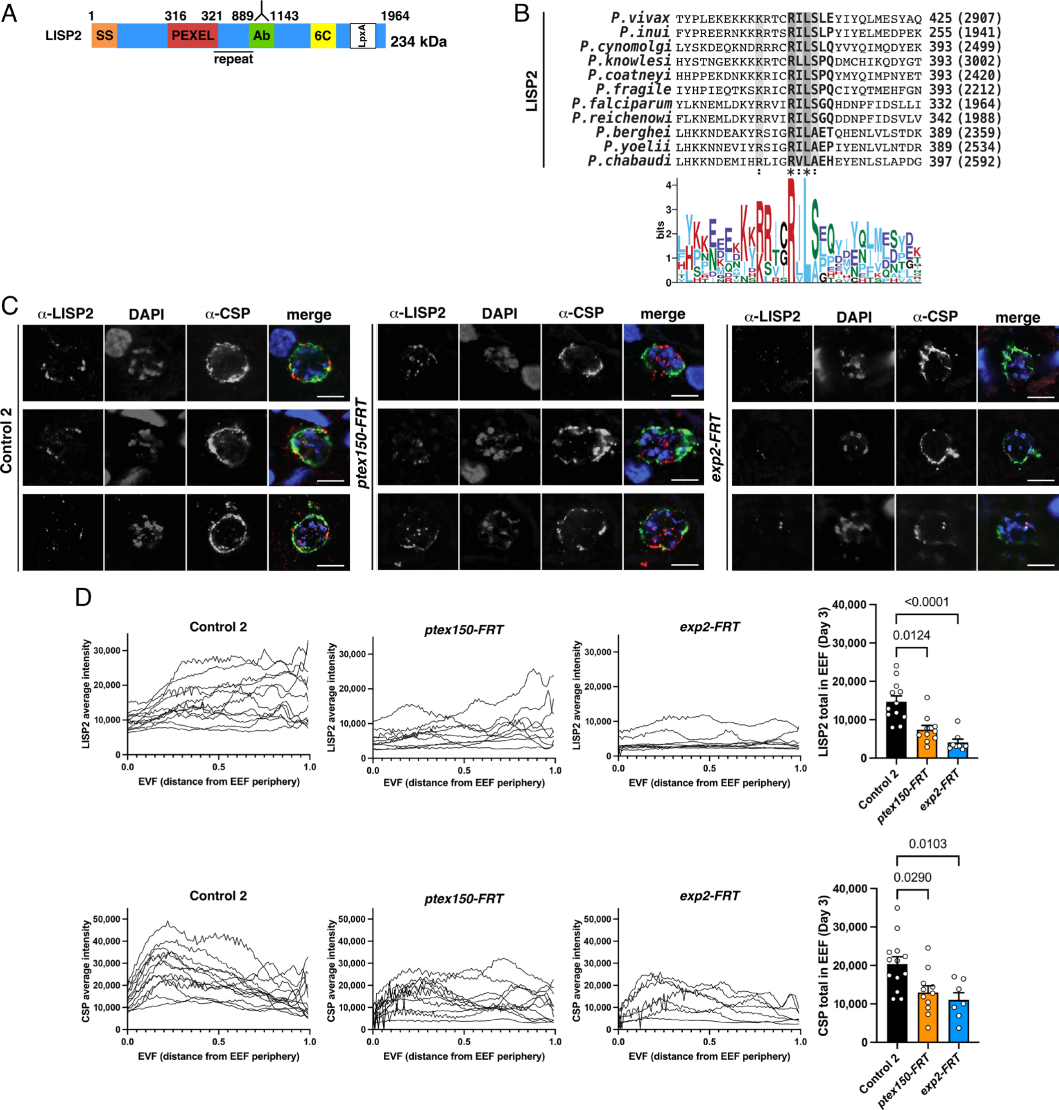
Localization of PEXEL proteins LISP2 and CSP in *P. falciparum* EEFs. (*A*) Schematic of *P. falciparum* LISP2 and LSA3 including SS, PEXEL, antibody binding region (Ab), 6C domain, LpxA domain, transmembrane domain (TM). (*B*) Multiple sequence alignment of LISP2 from *Plasmodium* species shows the PEXEL motif is conserved. (*C*) Immunofluorescence microscopy of LISP2 in *P. falciparum* Control 2, *ptex150-FRT,* and *exp2-FRT* EEFs on day 3 postinfection. (Scale bar, 5 mm.) (*D*) Quantification of LISP2 (*Top*) and CSP (*Bottom*) pixel intensity over distance from the parasite periphery in *P. falciparum* Control 2, *ptex150-FRT,* and *exp2-FRT* EEFs on day 3 postinfection. Data are mean ± SEM from n = 7 to 13 individual EEFs per condition analyzed by one-way ANOVA (Kruskal–Wallis test). *P* values are shown; ns, not significant.

Having established that PTEX150 and EXP2 are important for normal growth of *P. falciparum* EEFs, we next sought to test the hypothesis that these proteins facilitate growth by enabling protein export into infected hepatocytes. This required identifying a liver stage exported protein, which has not yet been reported in *P. falciparum*. In *P. berghei*, LISP2 is N-terminally processed and exported to the hepatocyte (33) and homologs are present in other *Plasmodium* species, including *P. falciparum* (Fig. 5*A*). Alignment of LISP2 amino acid sequences identified a conserved PEXEL (Fig. 5*B*): The human- and primate-infecting species possessed a relaxed PEXEL (RxLxxE/Q/D) (10, 12), whereas the rodent-infecting species has a canonical motif. *P. falciparum* LISP2 contained the motif RILSGQ located 316 residues from the N terminus, further C-terminal than previously identified PEXEL motifs (3, 4, 10). However, the PEXEL position in other species is located a similar distance C-terminal to the signal peptide and is N-terminally processed in *P. berghei* (33) including after the conserved leucine residue (35). Therefore, the noncanonical position of the PEXEL still permits processing of the motif.

To test the idea that LISP2 is cleaved specifically by plasmepsin V, we incubated peptides containing the PEXEL sequences from *P. falciparum* and *P. berghei* LISP2 with recombinant plasmepsin V from *P. vivax* (62). Cleavage of LISP2 peptides occurred at a comparable rate to processing of the control peptide from knob-associated histidine-rich protein (KAHRP) while mutation of the conserved PEXEL arginine and leucine amino acids inhibited their cleavage altogether (*SI Appendix*, Fig. S4*A*). This demonstrated that, like KAHRP, LISP2 peptide processing was possible by plasmepsin V and was PEXEL dependent. These results are consistent with *P. falciparum* and *P. berghei* LISP2 containing a PEXEL sequence that can be processed by the protease involved in the export of multiple proteins during the blood stage (7, 8).

We next investigated the subcellular localization of LISP2 in *P. falciparum*–infected hepatocytes from sections of livers from humanized mice. We generated an antibody to *P. falciparum* LISP2 at a position N-terminal to the 6-Cys domain (6C), incorporating part of the repeat region (green box, Fig. 5*A*). The antibody localized in puncta at the periphery of EEFs by IFA, sometimes internal to, or colocalizing with, anti-CSP antibodies, suggesting that it was secreted to the parasite periphery, likely including the PVM, on days 3 and 5 postinfection, while on day 6 also being present inside the parasite and at the periphery (Fig. 5*C* and *SI Appendix*, Fig. S4*B*). Unlike in *P. berghei* (33), we did not detect the immunoreactive LISP2 fragment localizing within the cytoplasm or nucleus of the *P. falciparum*–infected hepatocyte, but rather it was at the EEF periphery. While this suggests that LISP2 may not be exported by *P. falciparum* EEFs, we cannot exclude that it was bound to the cytoplasmic face of the PVM following export or that another fragment of LISP2 is exported into the cytoplasm that was not recognized by our antibody. Interestingly, antibodies to the C terminus of *P. vivax* LISP2 showed that this fragment also localized at the EEF periphery (63), suggesting that species-specific LISP2 trafficking may occur. Quantification of LISP2 and CSP pixels in *ptex150-FRT* or *exp2-FRT* EEFs from IFAs of independent liver sections indicated that both proteins were expressed at lower levels compared to Control 2 EEFs on day 3 postinfection, (Fig. 5*D*). It was not possible to determine whether this reduction was linked to PTEX loss-of-function or was a secondary effect of parasites being unfit and/or dying. Altogether, based on IFAs with our antibody, we were unable to test our hypothesis that PTEX is required for export of LISP2 into the *P. falciparum*–infected hepatocyte.

As described earlier, we also encoded an ectopically expressed PbLISP2-NLS-HA reporter in our *ptex*-*FRT* constructs for assessment of protein export by *P. falciparum* (Fig. 2*A* and *SI Appendix*, Fig. S5*A*). IFAs of PbLISP2-NLS-HA during the blood stage indicated the protein was expressed at low levels in rings and trophozoites and more strongly in schizonts (*SI Appendix*, Fig. S5*B*). Immunoblots indicated that PbLISP2-NLS-HA migrated circa 40 kDa, indicating the protein was N-terminally processed at a site C-terminal of the signal sequence (SS), suggestive of PEXEL processing (*SI Appendix*, Fig. S5*C*). However, the mass was larger than predicted (*SI Appendix*, Fig. S5*A*), a feature of some proteins due to posttranslational modification, unusual amino acid sequences, and/or biochemical properties (64). Consistent with this, LISP2 also migrated aberrantly in *P. berghei*–infected cells (33). Subcellular fractionation of asexual parasites and immunoblotting revealed that PbLISP2-NLS-HA was present in both the pellet and supernatant following saponin or equinatoxin treatment, indicating the protein was distributed across the PV, PVM, and host erythrocyte (*SI Appendix*, Fig. S5*C*). While equinatoxin treatment suggested that ~30% of the protein was exported, like the REX3 control, this could not be clearly visualized by microscopy (*SI Appendix*, Fig. S5*B*). Similarly, while expression of PbLISP2-NLS-HA was detected by IFA in *P. falciparum*–infected hepatocyte sections from humanized mice on day 5 postinfection, we only observed expression inside EEFs and sometimes at the periphery, but not within the infected hepatocyte nucleus or cytoplasm (*SI Appendix*, Fig. S5*D*), and we did not detect its expression in livers isolated from mice on day 3. We therefore could not use PbLISP2-NLS-HA to answer whether PTEX was required for protein export in *P. falciparum* EEFs.

### Antibodies to Liver-Stage Antigen 3 (LSA3) Localize within the Parasite and at the Periphery of *P. falciparum* EEFs.

**Fig. 6.**
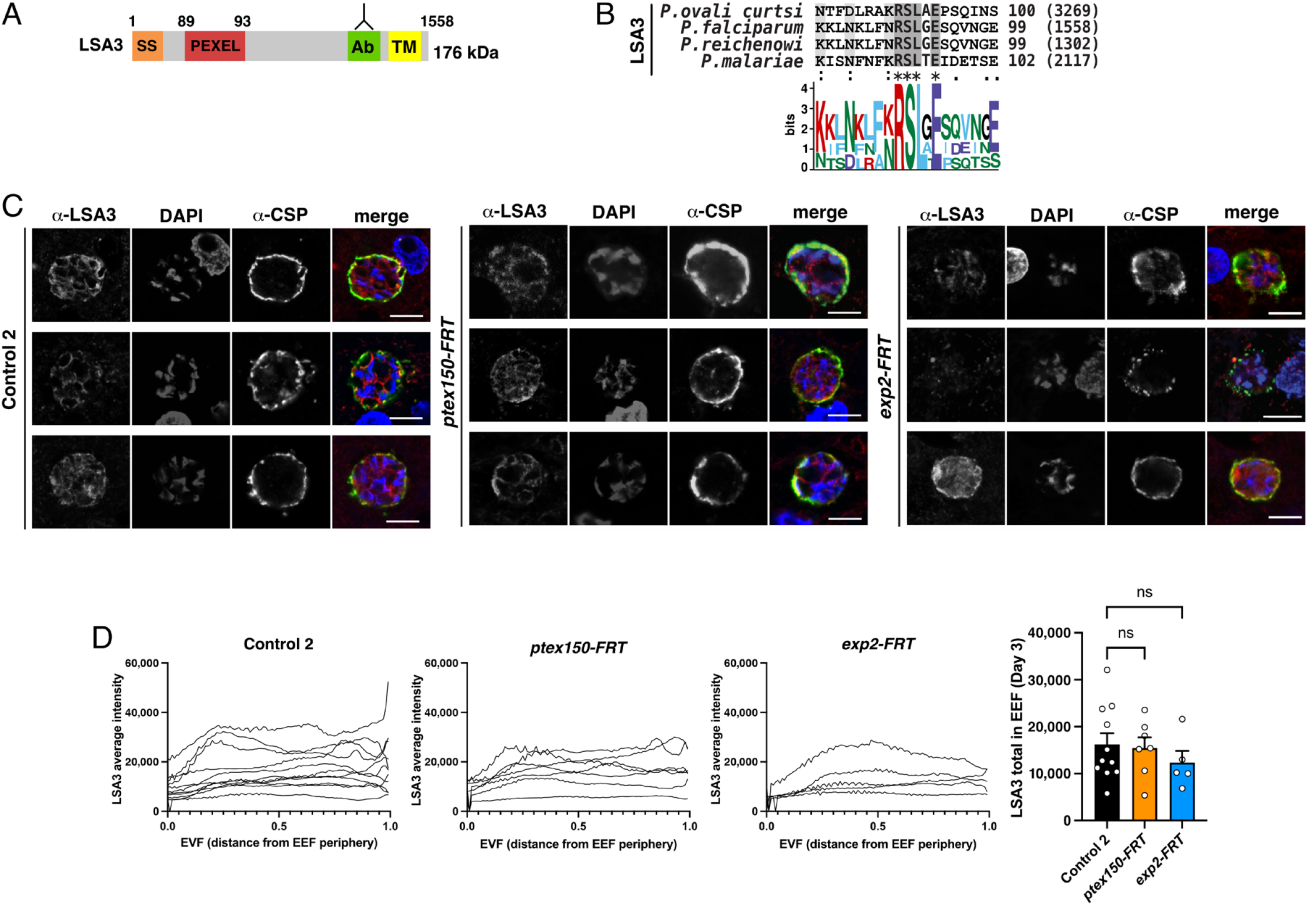
Localization of PEXEL protein LSA3 in *P. falciparum* EEFs. (*A*) Schematic of *P. falciparum* LSA3 including SS, PEXEL, Ab region, TM domain. (*B*) Multiple sequence alignment of LSA3 from *Plasmodium* species shows the PEXEL motif is conserved. (*C*) Immunofluorescence microscopy of LSA3 in *P. falciparum* Control 2, *ptex150-FRT,* and *exp2-FRT* EEFs on day 3 postinfection. (Scale bar, 5 mm.) (*D*) Quantification of LSA3 distance from the parasite periphery in *P. falciparum* Control 2, *ptex150-FRT,* and *exp2-FRT* EEFs on day 3 postinfection. Data are mean ± SEM from n = 5 to 11 individual EEFs per condition analyzed by one-way ANOVA (Kruskal–Wallis test). *P* values are shown; ns, not significant.

LSA3 contains a PEXEL motif (65) and is expressed by *P. falciparum* EEFs (66) (Fig. 6*A*). Alignment of LSA3 amino acid sequences from *Plasmodium* spp. indicated that the PEXEL motif is conserved in all species that contain an orthologous gene (Fig. 6*B*). We investigated the localization of LSA3 in *ptex150-FRT*, *exp2-FRT,* and Control 2 EEFs from livers of humanized mice by IFA. Antibodies targeting the C-terminal portion of LSA3 (65) localized in puncta inside the parasite and sometimes at the periphery labeled with CSP-specific antibodies, indicating LISP2 was secreted from the parasite to the periphery suggestive of the PV/PVM, but no export into the hepatocyte cytoplasm or nucleus was evident using the reagent available (Fig. 6*C*). Quantification of LSA3 in *ptex150-FRT* and *exp2-FRT* EEFs from IFAs of independent liver sections indicated that it was expressed at similar levels to Control 2 EEFs on day 3 postinfection (Fig. 6*D*). The localization of LSA3 to the PV/PVM in *P. falciparum* EEFs was consistent with a previous report (66); however, this peripheral localization precluded characterizing the role of PTEX in export by EEFs (Fig. 6).

### CSP Localizes to the Periphery of *P. falciparum* Liver Stages.

Our localization studies utilized CSP as a marker to readily identify EEFs in liver sections. CSP contains two PEXEL motifs and has previously been localized at the hepatocyte nucleus following *P. berghei* infection and in the erythrocyte cytoplasm when expression of the CSP N terminus was induced at the blood stage (32). However, deletion of the N-terminal domain of CSP that included both PEXEL sequences had no impact on liver-stage infection, suggesting that the PEXEL motifs are not essential (67). Interestingly, CSP localizes to the parasite periphery in *P. falciparum*–infected hepatocytes, including partial colocalization with ETRAMP10.3 by IFA (47). In our IFAs from humanized mice, CSP was not observed within the host cell or nucleus but partly colocalized with PTEX150, EXP2, and EXP1 on days 3, 5, and 6 postinfection (e.g., Figs. 1, 4, 5, and 6), suggesting that it was localized at the parasite membrane (PM) and/or PVM. We cannot exclude that some of the protein was located at the PM as confocal microscopy alone is not sufficient to discriminate between the PM and PVM. The lack of CSP export in our study and that previously (47) raised the question of whether CSP can be processed by plasmepsin V (67, 68). Incubation of synthetic CSP peptides containing the first or second PEXEL motif with plasmepsin V and mass spectrometry analyses revealed processing of both CSP peptides after the conserved PEXEL leucine residue (*SI Appendix*, Fig. S6). Therefore, CSP possesses sequences that plasmepsin V can cleave biochemically. Whether the CSP PEXEL motifs have an important function during other lifecycle stages, such as sporozoites, is unclear (67). Altogether, these results show that CSP localizes to the periphery of *P. falciparum* EEFs despite possessing two PEXEL motifs that can be cleaved by plasmepsin V in a biochemical assay. The localization of PEXEL proteins CSP, LISP2, and LSA3 to the periphery of *P. falciparum* EEFs (*SI Appendix*, Table S1) thereby precluded further characterization of PTEX translocation function in hepatocytes by IFA.

## Discussion

Few studies of essential blood-stage proteins have extended to liver stages of *P. falciparum* due to limited tools for conditionally regulating protein expression in EEFs. The rapamycin-inducible DiCre-*lox* system was elegantly employed in *P. falciparum* gametocytes and also following sporozoite invasion of hepatocyte culture, where excision was detectable 72 h later, but mutations were not generated for functional studies in early liver stages (69). Here, we used FlpL/*FRT* (49, 50) to autonomously excise genes in *P. falciparum* during sporogony for study across the liver stage. A similar approach was used in *P. berghei* sporozoites, though the 3′ untranslated region (UTR) of genes was targeted to knockdown gene expression (46, 49, 70, 71). We placed *FRT* sequences within native or surrogate introns, allowing excision of protein coding sequences and thus the generation of conditional knockout parasites. The FlpL/*FRT* system was efficient at targeting *ptex150* and *exp2* during mosquito passage and should be amenable to other *P. falciparum* genes for pre-erythrocytic functional genomics studies.

Like BS, liver stages reside within a PVM, alter the infected cell, and express components of the protein export pathway. Given the importance of protein export for asexual blood-stage growth, this pathway represents an important pre-erythrocytic target (29). Yet functional characterization of protein export in the *P. falciparum* liver stage has remained unreported. The rodent-infecting parasite *P. berghei* has provided knowledge of this aspect of parasite biology. *P. berghei* EEFs express PTEX components EXP2, PTEX88, and TRX2 but apparently not HSP101, while the status of PTEX150 remains unknown (42, 46, 72). The absence of HSP101 from *P. berghei* EEFs is fascinating as it is the ATPase for unfolding cargo for transport across the PVM (15–17). Overexpression of HSP101 in *P. berghei* EEFs did not prompt the transport of PEXEL proteins beyond the PVM (40), suggesting that this is not the sole factor limiting export into the hepatocyte. However, the apparent absence of HSP101 from *P. berghei* EEFs provides further rationale to characterize PTEX in *P. falciparum* liver stages. A recent study demonstrated expression of HSP101 at the periphery of *P. falciparum* EEFs (73) and a function during the liver stage remains plausible. The FlpL/*FRT* system adapted herein provides an ideal approach to address the functional importance of HSP101 in liver stages.

Previously, it was shown that *P. falciparum* EEFs express PTEX150 and EXP2 at the periphery on day 5 pi (47). Here, we showed that both are expressed on days 3, 5, and 6 postinfection, suggesting that they play a function in EEFs earlier than previously appreciated. PTEX150 and EXP2 partly colocalized with EXP1, suggesting that they were at the PV/PVM, consistent with their localization in infected erythrocytes (15) and hepatocytes (47) and therefore a putative role in EEF protein export. PTEX150- and EXP2 deficiency reduced the fitness of *P. falciparum* EEFs with clearance from the liver evident by day 3 postinfection, suggesting that both proteins are important during early stages of hepatocyte infection, as well as in later stages. Conditional knockdown of *P. berghei* EXP2 using the *glmS* ribozyme in cultured hepatocytes also caused a fitness cost to EEFs (42) demonstrating a conserved, essential function for EXP2 in *Plasmodium* replication in hepatocytes. EXP2 has two roles in the blood stage, as a PVM nutrient channel and also a pore for protein export (18, 21). It is possible that EXP2 supports both nutrient import and protein export functions during the liver stage. Interestingly, while some PTEX150-deficient EEFs were cleared from the livers of humanized mice, a significant proportion remained as late as day 6 postinfection. While this suggests that PTEX150 may not be as critical as EXP2, humanized mice lack B and T cells capable of eliminating infected human hepatocytes (74). This may contribute to the persistence of PTEX150-deficient EEFs in the livers of humanized mice that may not occur in a fit vertebrate host.

It is tempting to speculate that PTEX deficiency confers a fitness cost due to a block of protein export. Testing this hypothesis will require the identification of a protein that *P. falciparum* exports beyond the PVM. We localized four PEXEL proteins using antibodies in order to limit the possibility that tags such as mCherry would trap the cargo at the PVM, which it did for LISP2 in *P. berghei* EEFs (33). While we cannot exclude that the peripheral localization observed for LISP2, LSA3, CSP, and PbLISP2-NLS-HA may involve PTEX translocation followed by binding to the cytoplasmic face of the membrane, we could not conduct a robust analysis of PTEX translocation function in hepatocytes via targeting to the cytosol.

The localization of multiple *P. falciparum* PEXEL proteins to the periphery of infected hepatocytes demonstrates the complexity of protein transport between blood and liver stages of this species, and such differences have been described previously. While the PEXEL motif facilitates export to the erythrocyte via plasmepsin V cleavage (5–8), targeting of PEXEL proteins to the PVM of infected hepatocytes is not without precedent in *P. berghei* (35, 39) or *P. falciparum* (48). It may be that parasites export a smaller subset of proteins into the hepatocyte than in the erythrocyte to avoid immune detection including via MHC class I, but as with *Toxoplasma*, it is clear that intracellular parasites also co-opt host cellular signaling from the PVM (75–77). The homolog of plasmepsin V in *Toxoplasma* processes several PEXEL substrates for targeting to the PV/PVM, while other *Toxoplasma* substrates are trafficked to the host cell (78–81) demonstrating similarities in N-terminal processing and targeting by these parasites (82).

LSA3 is an antigenic liver-stage protein that was localized previously to the PVM (66). The function and importance of LSA3 remains unknown; however, we localized it during hepatocyte infection using a more recently developed antibody (65), which confirmed a PVM localization in the hepatocyte. Interestingly, PfLISP2 and the reporter PbLISP2-NLS-HA also localized to the EEF periphery but not in the host cell cytosol or nucleus. This was unexpected, as *P. berghei* efficiently exports LISP2, but this protein is processed multiple times and the exported fragment remains to be mapped (33); it is possible that our antibodies did not recognize the exported fragment of PfLISP2 or alternatively that the NLS intended to concentrate PbLISP2-NLS-HA in the host cell nucleus affected trafficking. The PEXEL of LISP2 from *P. berghei* and *P. falciparum* is located further from the N terminus than other exported proteins (10). We showed that this unusual position is conserved in LISP2 from other *Plasmodium* species and that peptides containing the PEXEL were efficiently processed by plasmepsin V, supporting the observations that LISP2 is N-terminally processed prior to export in *P. berghei*–infected hepatocytes (33, 35) likely by this protease.

Our observation that CSP localized to the EEF periphery/PVM contrasted with an earlier study in which *P. berghei* CSP was exported in a PEXEL-dependent manner (32). However deletion of the CSP N terminus including both PEXEL sequences in *P. berghei* did not affect liver-stage development (67), and antigen presentation of CSP by infected hepatocytes does not require functional PEXEL motifs (36) indicating these motifs are probably not critical (67). The conservation of PEXELs of CSP in multiple *Plasmodium* species raises the question of their functional importance. Mass spectrometry analyses of *P. falciparum* and *P. berghei* sporozoites showed abundant CSP expression with all peptides mapping C-terminal of the first PEXEL motif (56–59, 83, 84). This suggests that processing of one or both PEXELs may occur in sporozoites; however, whether this is physiologically important is unknown.

In summary, we employed Flp/*FRT* to disrupt PTEX150 and EXP2 expression in *P. falciparum* EEFs. Our results indicate that both PTEX members are important for EEF growth and suggest that EXP2 may have two functions, as it does at the blood stage. We also identified proteins with PEXEL motifs that are targeted to the periphery of infected hepatocytes, likely at the PVM, in agreement with *P. berghei* studies, suggesting that the *P. falciparum*–hepatocyte interface contains exported proteins. PVM targeting may be a mechanism by which EEFs regulate host cellular processes while limiting antigen presentation.

## Methods

### Parasites, Mosquitoes, Hepatocyte Assays, Humanized Mice, and Statistics.

Methods for *P. falciparum* culture, mutagenesis, mosquito transmission, hepatocyte culture and infection assays, humanized mouse infections, and statistics are explained in detail in *SI Appendix*, *SI Methods* section.

### Microscopy, Immunoblots, CSP Processing, and Mass Spectrometry.

Methods for immunofluorescence microscopy, immunoblotting, and plasmepsin V cleavage assays are explained in detail in *SI Appendix*, *SI Methods* section.

## Supplementary Material

Appendix 01 (PDF)

## Data Availability

All study data are included in the article and/or *SI Appendix*.
