# Supplementary material for: Flp/FRT-mediated disruption of ptex150 and exp2 in Plasmodium falciparum sporozoites inhibits liver-stage development
Source: Proc Natl Acad Sci U S A. 2024 Jul 5;121(28):e2403442121. doi: 10.1073/pnas.2403442121 (PMC11252984; doi:10.1073/pnas.2403442121)
Supplement: Supplementary file 1 — Appendix 01 (PDF) [file pnas.2403442121.sapp.pdf]

Supporting Information for
Flp/*FRT*-mediated disruption of *ptex150* and *exp2* in *Plasmodium falciparum* sporozoites inhibits liver stage development

Robyn McConville[1,2], Jelte M.M Krol[1,2], Ryan W.J. Steel[1,2], Matthew T. O'Neill[1], Bethany K. Davey[1,2], Anthony N. Hodder[1,2], Thomas Nebl[1,2], Alan F. Cowman[1,2], Norman Kneteman[3] and Justin A. Boddey[1,2,*]

Justin A. Boddey
Email: boddey@wehi.edu.au

This PDF file includes:

Figures S1 to S6
Tables S1 to S2
SI Methods
SI References

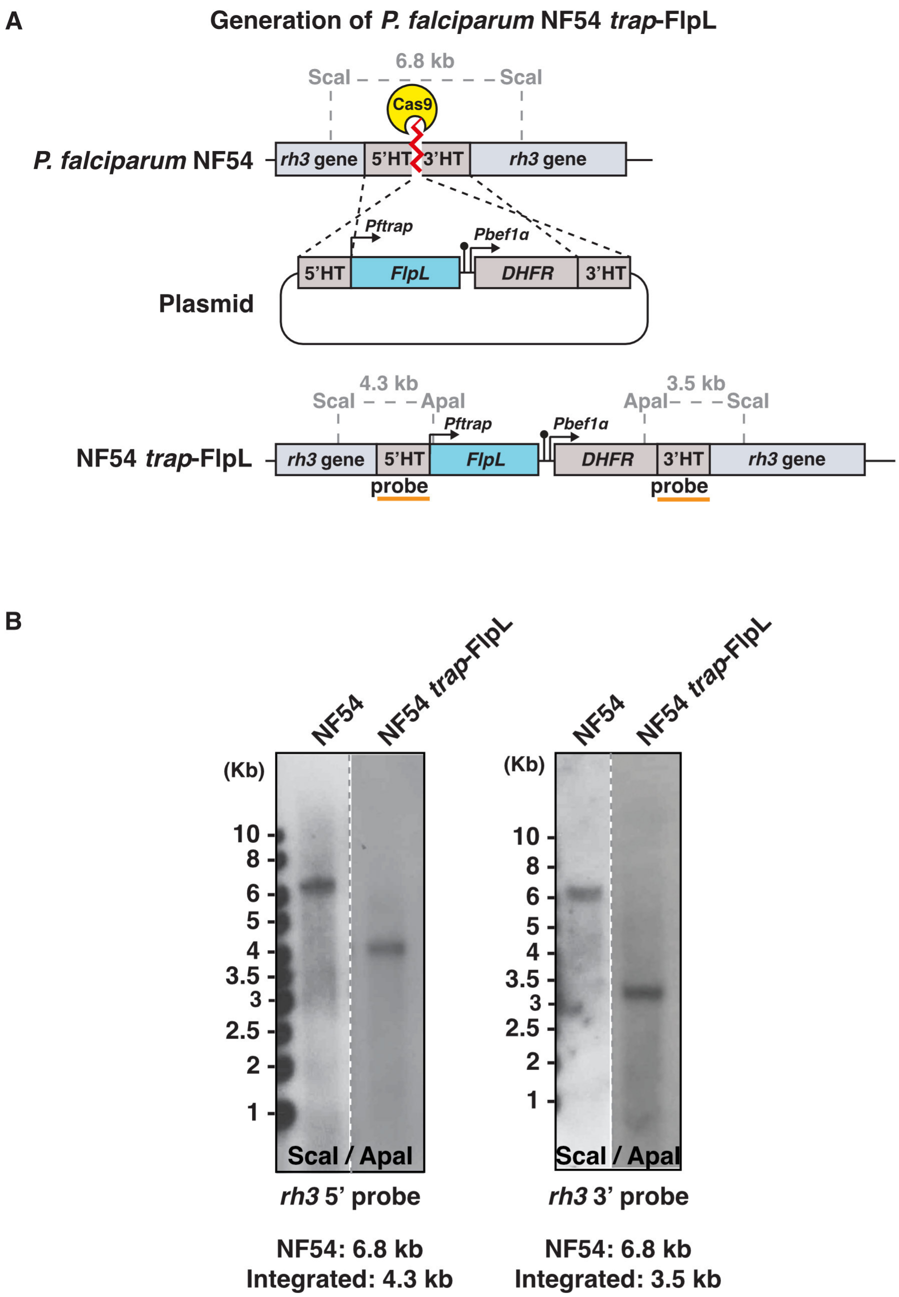

Fig. S1. Generation of *P. falciparum* NF54 *trap*-FlpL parasites. (*A*) Strategy for inserting the FlpL recombinase expression cassette (*Pftrap* gene promoter-FlpL recombinase-*Pftrap* gene terminator) into the *P. falciparum rh3* pseudogene by double cross-over homologous recombination. HT, homology target. (*B*) Southern blot analysis of parental NF54 and NF54 *trap*-FlpL integrant parasites after digestion of genomic DNA with *Sca*I/*Apa*I. The probes used (orange bars) for genotyping and the expected fragment sizes after digestion are shown. Data represent n=1 experiment confirming integration.

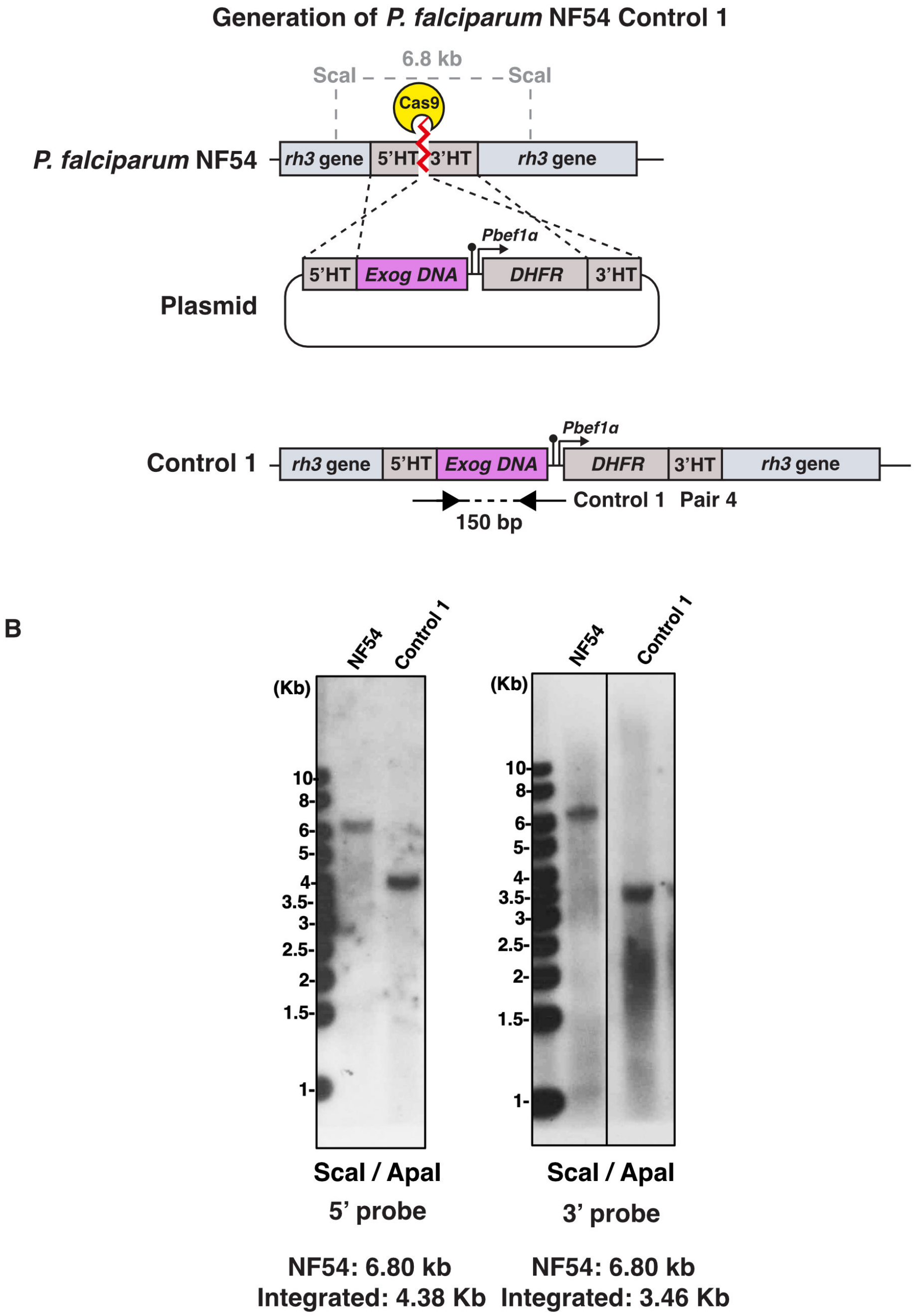

Fig. S2. Generation of *P. falciparum* NF54 Control 1 parasites. (*A*) Strategy for inserting a small exogenous DNA fragment (Exog DNA) into the *P. falciparum rh3* pseudogene by double cross-over homologous recombination. HT, homology target. (*B*) Southern blot analysis of parental NF54 and Control 1 integrant parasites after digestion of genomic DNA with *Sca*I/*Apa*I. The probes used were the same as in Fig. S1. and the expected fragment sizes after digestion are indicated. Data represent n=1 experiment confirming integration.

Generation of *P. falciparum* NF54 Control 2

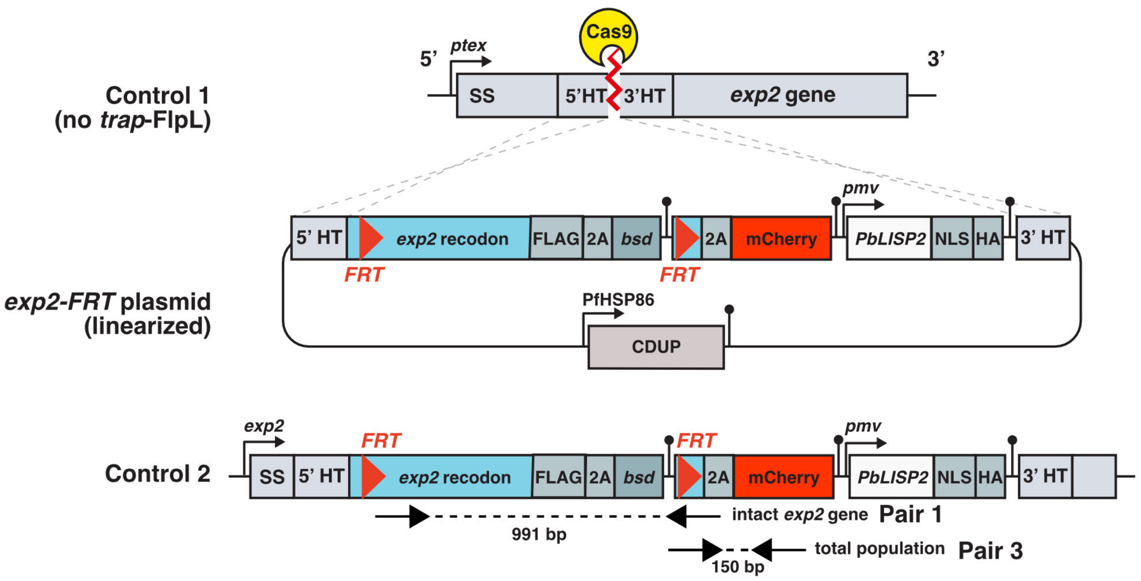

Fig. S3. Generation of *P. falciparum* NF54 Control 2 parasites. Strategy to integrate the *exp2-FRT* plasmid into NF54 Control 1 parasites lacking the *trap*-FlpL cassette by double cross-over homologous recombination. The construct contained blasticidin deaminase (bsd) for positive selection and cytosine deaminase (CDUP) for negative selection. HT, homology target; 2A, skip peptide.

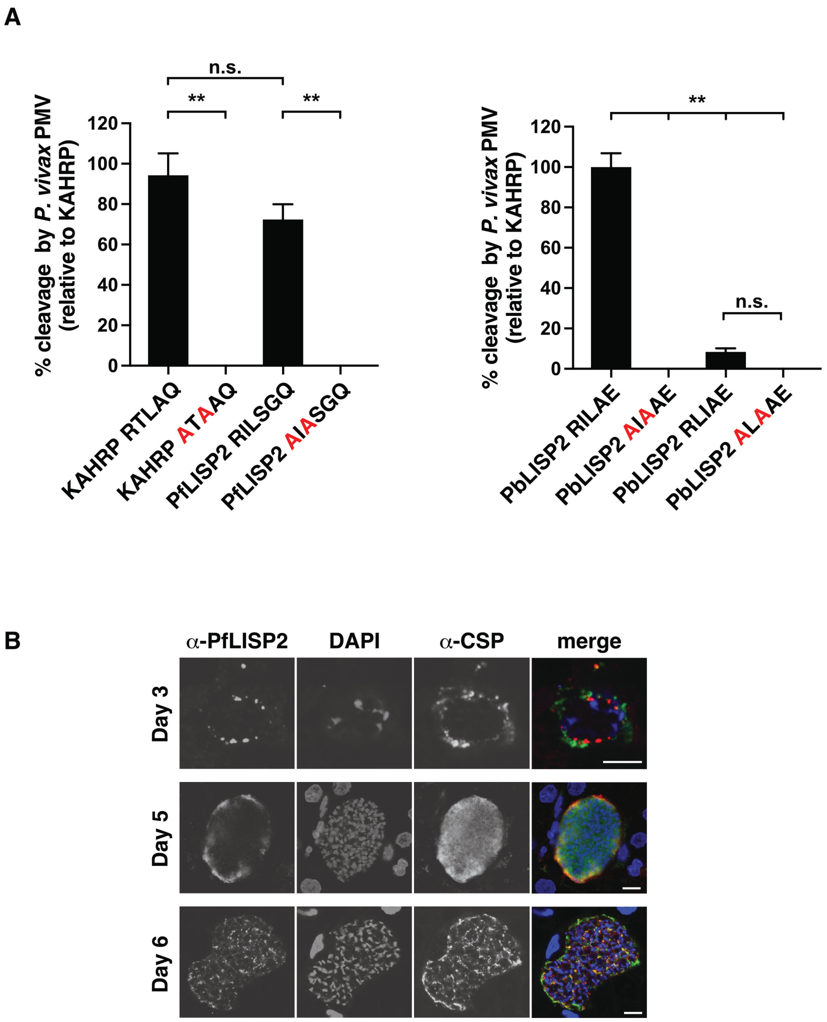

Fig. S4. LISP2 peptide processing by plasmepsin V and localization in *P. falciparum* EEFs. (*A*) Peptides of KAHRP (*P. falciparum*), PfLISP2 and PbLISP2 containing a PEXEL motif are cleaved by recombinant plasmepsin V (PMV) from *P. vivax*. Mutation of the conserved PEXEL arginine and leucine residues to alanine (red) inhibited cleavage. Data are mean and error bars represent s.e.m. from n=3 pooled independent experiments. (*B*) Immunofluorescence microscopy of fixed liver sections isolated from humanized mice reveals the expression and subcellular localization of PfLISP2 in *P. falciparum* EEFs on days 3, 5 and 6 post-infection (refer to Fig. 5). Scale bar, 5 um.

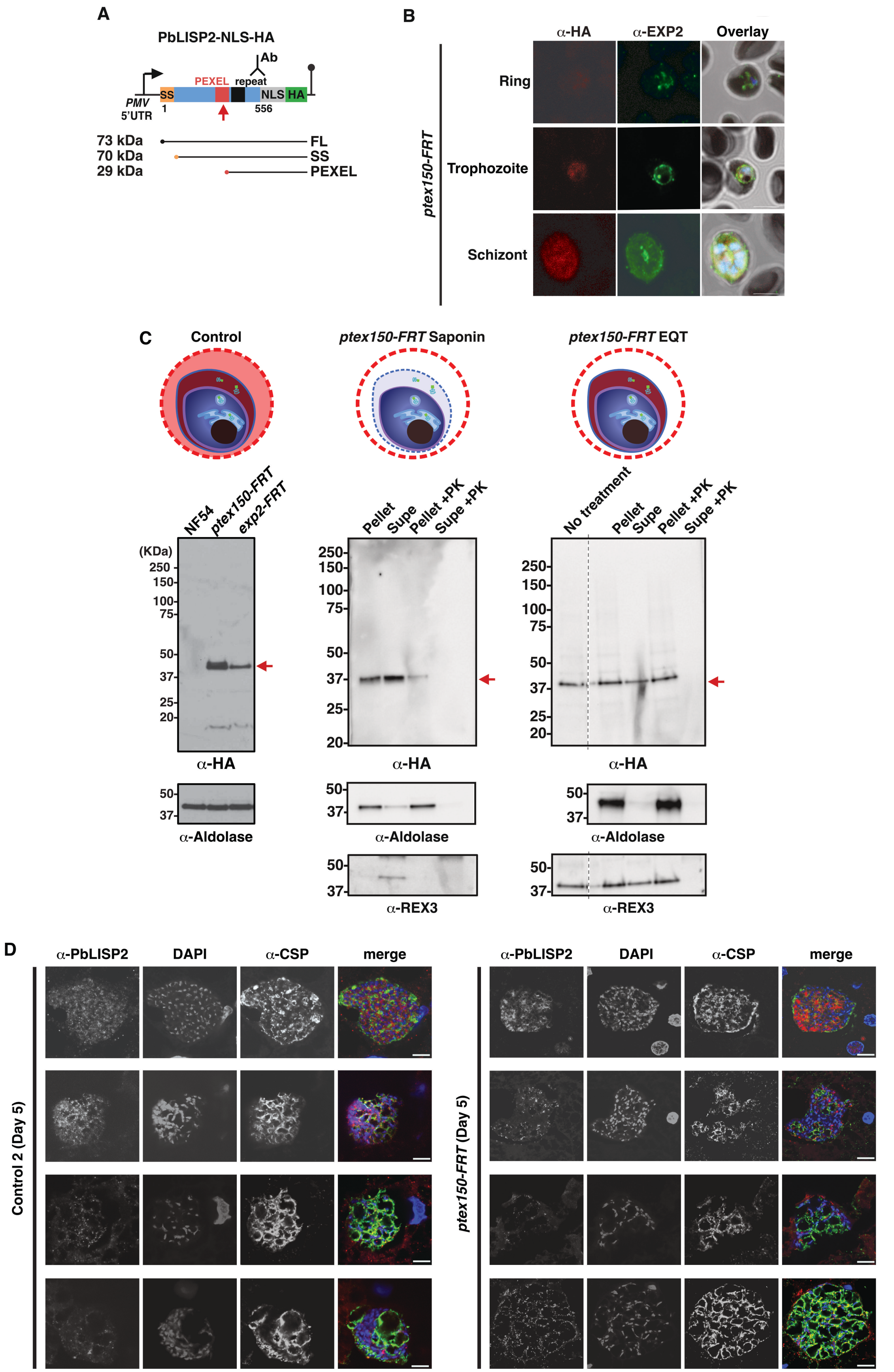
A
PbLISP2-NLS-HA
Ab
PEXEL
repeat
SS
NLS
HA
PMV
5'UTR
1
556
73 kDa
70 kDa
29 kDa
FL
SS
PEXEL
B
α-HA
α-EXP2
Overlay
ptex150-FRT
Ring
Trophozoite
Schizont
C
Control
ptex150-FRT Saponin
ptex150-FRT EQT
NF54
ptex150-FRT
exp2-FRT
(KDa)
Pellet
Supe
Pellet +PK
Supe +PK
No treatment
α-HA
α-Aldolase
α-REX3
D
α-PbLISP2
DAPI
α-CSP
merge
Control 2 (Day 5)
ptex150-FRT (Day 5)

Fig. S5. Expression, processing, and localization of PbLISP2-NLS-HA during *P. falciparum* infection of erythrocytes and hepatocytes. (*A*) Schematic of the mini *P. berghei* LISP2-NLS-HA reporter, comprising the first 556 residues of PbLISP2 (PBANKA_1003000) containing the signal sequence (SS), canonical PEXEL (RILAE) and a single copy of the non-canonical PEXEL RLIAE from the repeat region fused to a nuclear localization sequence (NLS) and triple hemagglutinin (HA) tag. Expression was driven from the *P. falciparum plasmepsin V* (*PMV*) gene promoter. The transgene was included in *ptex-FRT* plasmids (see Fig. 2A). (*B*) Immunofluorescence microscopy with anti-HA antibodies shows expression of PbLISP2-NLS-HA in *P. falciparum ptex150-FRT* asexual blood stages with little or inefficient export to the host cell. EXP2 marks the PVM. Scale bar, 5 um. (*C*) Control: Immunoblot with anti-HA antibodies shows expression and N-terminal processing of PbLISP2-NLS-HA in *P. falciparum ptex150-FRT- and exp2-FRT*-infected erythrocytes. HA-specific bands have a size indicative of processing by plasmepsin V within the PEXEL motif (red arrows). Aldolase was included as a control. *ptex150-FRT* Saponin: Immunoblot of erythrocytes infected with *ptex150-FRT* parasites treated with 0.15% saponin to permeabilize the RBC membrane and PVM, centrifuged, and pellet and supe fractions treated with or without proteinase K (PK; 50 ug/ml) before separation by SDS-PAGE. PbLISP2-NLS-HA was partially liberated into the saponin supernatant (supe) and was susceptible to PK, indicating it was within the PV and PVM. Aldolase (cytoplasm) was included to assess integrity of the parasite membrane. *ptex150-FRT* EQT: Immunoblot of erythrocytes infected with *ptex150-FRT* parasites treated with equanatoxin II (EQT; 5 ug) to permeabilize the RBC membrane (leaving PVM intact), centrifuged, and pellet and supe fractions treated with or without PK before separation by SDS-PAGE. PbLISP2-NLS-HA was partially liberated into the EQT supe and was susceptible to PK, indicating it was partially (~30%) exported like the exported REX3 control. (*D*) Immunofluorescence microscopy of humanized mouse liver sections infected with Control 2 or *ptex15-FRT* parasites using anti-PbLISP2 antibodies shows expression of PbLISP2-NLS-HA in *P. falciparum* liver-stages in humanized mice on day 5 post-infection. The protein was located predominantly inside EEFs and was not exported into the hepatocyte nucleus or to the host cell cytoplasm efficiently. HA antibodies were not used due to high cross-reactivity background labelling of hepatocytes. Unexpectedly, no expression with anti-PbLISP2 antibodies was detected on day 3 post infection. Scale bar, 10 um.

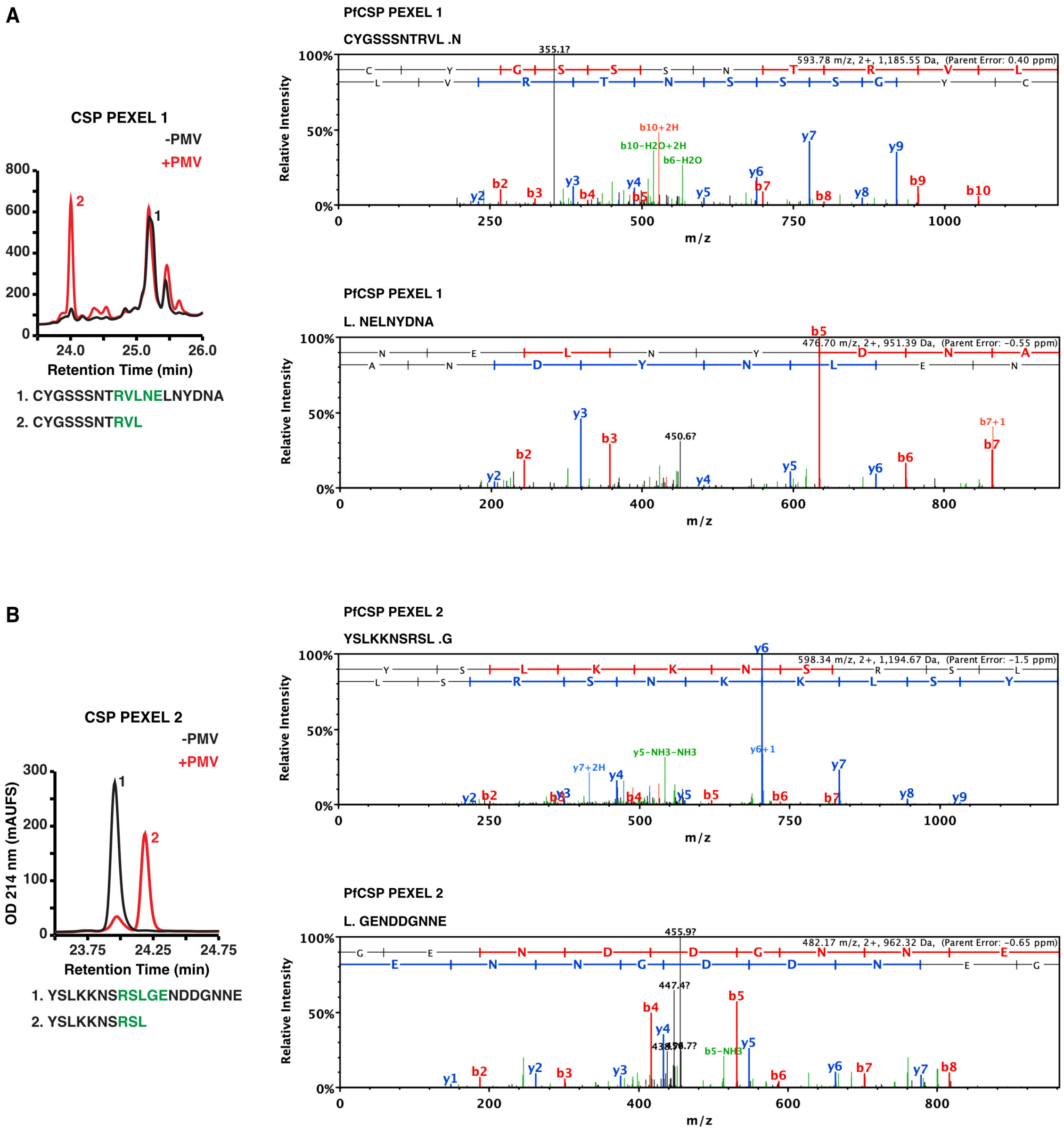

Fig. S6. ***P. falciparum*** **CSP contains two PEXEL motifs that are cleaved by plasmepsin V.** (*A*) Left: RP-HPLC shows that uncleaved peptides containing PEXEL motif P1 from *P. falciparum* CSP (-PMV; black) are cleaved in the presence of *P. falciparum* plasmepsin V-HA-agarose (+PMV; red). Right: LC-MS/MS identification of CSP peptide 1 cleavage products following incubation with plasmepsin V-HA-agarose. Peptide CYGSSSNT**RVLNE**LNYDNA was cleaved to CYGSSSNT**RVL**↓(top) and ↓**NE**LNYDNA (bottom). (*B*) Left: RP-HPLC shows that uncleaved peptides containing PEXEL motif P2 from *P. falciparum* CSP (-PMV; black) are cleaved in the presence of *P. falciparum* plasmepsin V-HA-agarose (+PMV; red). Right: LC-MS/MS identification of CSP peptide 2 cleavage products following incubation with plasmepsin V-HA-agarose. Peptide YSLKKNS**RSLGE**NDDGNNE was cleaved to YSLKKNS**RSL**↓ (top) and ↓**GE**NDDGNNE (bottom).

Table S1. Subcellular localization of proteins in *P. falciparum*-infected cells from this study

**Protein** (Gene ID)	**Family**	**Localization Pf blood stage**	**Localization Pf liver stage**	**Comment**	**References**
**EXP1** (PF3D7_1121600)	PVM protein	PVM	PVM	PVM-resident protein that aids EXP2-mediated nutrient transport	(1-4), this study
**PTEX150** (PF3D7_1436300)	PTEX component	PVM	PVM	Translocon component at PVM for protein export	(5-9), this study
**EXP2** (PF3D7_1471100)	PTEX component	PVM	PVM	Translocon pore at PVM for protein export<br>Also functions as a PVM nutrient channel independent of PTEX	(5, 9-13), this study
**PfLISP2** (PF3D7_0405300)	PEXEL	Not tested	EEF periphery likely PV / PVM	Function unknown<br>PfLISP2 peptides are cleaved by plasmepsin V	(14, 15), this study
**LSA3** (PF3D7_0220000)	PEXEL	PVM	EEF periphery likely PV / PVM	Immunogenic protein. Cleaved by plasmepsin V in asexual blood stage	(16, 17), this study
**CSP** (PF3D7_0304600)	PEXEL	Not tested	EEF periphery likely PV / PVM	Essential for sporozoite development, motility and infectivity. Role in liver stage not clear<br>CSP-GFP reporter cleaved by plasmepsin V in blood stage	(18-20), this study
**PbLISP2** (PBANKA_1003000)	PEXEL	Not tested.<br>*pmv*-PbLISP2-HA reporter localizes to PV, PVM and host cell	EEF	Required for exo-erythrocytic merogony<br>PEXEL cleaved in merosomes<br>PbLISP2 peptides are cleaved by plasmepsin V	(21, 22), this study

PV, parasitophorous vacuole; PVM, parasitophorous vacuole membrane; EEF, exoerythrocytic form;
Pb, *P. berghei*; Pf, *P. falciparum*.

Table S2. Oligonucleotide primers used in this study

**Primer name**	**Sequence**
FLP01	CCGCTCGAGTATTTTTACAATTTTAATAATAATACTAAAAC
FLP02	TCCCCGCGGCTTCTGAGGGAATTATTTTTTTTTTGGTAAATT
FLP03	TCCCCCCGGGATATAATACTTGAATAATATTTTATGTAATATAGTG
FLP04	AAACTGCAGGGAAAATTTTTTCTATATATTATTTTAAATATGTATG
FLP05	AAAGCGGCCGCGAAGATGTTGATTTTAGGTACTATGTC
FLP06	ACCTTAATTACCCATTTCTGTATTACCAATACGCCC
FLP07	ATCCCTAGGATGCGGAAGCAATAAATGTGGTTTTAGAAG
FLP08	ATCGGCGCCGGTTTCTTTCTCTTCTATAATATTTTTTATATTATTCATCC
**q-RTPCR primers**	**Sequence**
WT_F	GGTAATACAGAAATGGTTAATCAATTCCGATGAC
WT_R	GGAACCTTTTTAATGCTTGCATTGCAGAATATC
*ptex150-FRT*_F	ATGTAATGCCCACCTGCAGCATGCCAGGGAACGATAGTGAT
ptex150-FRT _R	CTCCGGCTTGTTTCAGCAGAGAGAAGTTTGTTGCGCCG
*exp2-FRT*_F	ATATAATATTATTTACTTCTACATCCACTGAAAAA
*exp2-FRT*_R	GGGTCCTTGATGACTGTGGT
18S_F	GTAATTGGAATGATAGGAATTTACAGGT
18S_R	TCAACTACGAACGTTTTAACTGCAAC

SI Methods

Parasites and transgenesis

P. falciparum NF54 (kindly provided by the Walter Reid Army Institute of Research) asexual stages were cultured in human O-positive erythrocytes (Melbourne Red Cross) at 4% haematocrit in RPMI-HEPES supplemented with 0.2% $NaHCO_3$, 7% heat-inactivated human serum (Melbourne Red Cross), and 3% AlbuMAX (ThermoFisher Scientific). Parasites were maintained at 37 °C in 94 % N_2, 5 % CO_2 and 1 % O_2. Gametocytes were cultured in RPMI-HEPES supplemented with 0.2 % $NaHCO_3$, and 10 % heat-inactivated human serum (Melbourne Red Cross). Gametocytes for transmission to mosquitoes were generated using the "crash" method (23) using daily media changes.

Generation of transgenic *P. falciparum*

Highly synchronized schizonts were enriched using magnet-activated cell sorting (MACS) magnetic separation columns (Miltenyi biotec) and incubated with E64 (Sigma) until parasitophorous vacuole (PV) enclosed merozoite structures (PEMS) could be visualized (24). Transfections were performed using Amaxa Basic Parasite Nucleofector Kit 2 (Lonza). Purified plasmid DNA (100 μg, Life Technologies) from each plasmid was transfected into *P. falciparum* NF54 and stable transfectants selected (25). Integration of plasmid DNA constructs was confirmed by Southern blot analysis using the Roche digoxigenin (DIG) system according to the manufacturer's instructions and diagnostic PCR.

The pCas9-PfPTEX plasmid was generated using a modified pUF-Cas9 vector that encodes both Cas9 and single guide RNA (sgRNA) (26, 27). The BtgZI adaptor sequence was replaced with the PTEX150 or EXP2 guide DNA sequences 5'- CCACCGTTATTAAAGATCCAATT -3' and 5'-CCTACTTGTAGTATGCCTGGAAA -3' (26). The BtgZI adaptor sequence was replaced with the PTEX150 or EXP2 guide DNA sequences 5'- CCACCGTTATTAAAGATCCAATT -3' and 5'-CCTACTTGTAGTATGCCTGGAAA -3' (26).

The FlpL recombinase construct was generated from the flpRD-CC-4 plasmid by replacing the *HSP86* promoter with the *TRAP* 5' UTR that had been inserted into the XhoI and SacII sites and amplified using primers FLP01 and FLP02 (Table S2). The *TRAP* 3' UTR was amplified by primers FLP03 and FLP04 and inserted into the construct using sites PacI and PstI in place of the *PbDT*-3' UTR. Finally, the *rh3* 5 and 3' homology target was amplified using primers FLP05 and FLP06 respectively and cloned into the plasmid using restriction enzymes PacI, NotI, and AvrII. The final construct was sequenced and linearized with StuI, PvuI and NotI and then transfected into NF54 *P. falciparum* parasites following standard procedures (28). Parasites that had correctly integrated the repair template were selected for after 10 days of incubation with 4 nM WR99210 (WR).

To enable FlpL/*FRT* conditional knockout of PTEX150 and EXP2, the constructs *PTEX150-FRT* and *EXP2-FRT* were synthesised by Epoch Life Science. The codon optimized *ptex* genes were fused to a FLAG epitope tag, a 2A skip peptide and the *blasticidin S deaminase* (*bsd*) gene to facilitate drug selection. A second 2A skip peptide fused to *mCherry* was incorporated to assess *FRT* recombination, such that excision by FlpL during mosquito passage placed *mCherry* in-frame with the truncated *PTEX* gene, producing a non-functional PTEX protein and the mCherry reporter. A chimeric PEXEL reporter protein was also incorporated in the *PTEX-FRT* constructs, comprising the first 354 amino acids of *P. berghei* liver specific protein 2 (PbLISP2) (21), containing the signal sequence and PEXEL motif, fused to the Hepatitis delta antigen nuclear localisation signal (NLS) followed by a triple hemagglutinin (HA) epitope tag. The rationale for directing PbLISP2-HA to the hepatocyte nucleus if exported was that this localization should increase the concentration, and extend the stability and half-life, of the reporter for microscopic visualization. The HA epitope would also allow use of commercial antibodies in addition to anti-PbLISP2 antibodies (21, 29) to detect expression and localize the reporter. The *cytosine deaminase* cassette was generated by digestion of the pCC1 plasmid with XhoI and PacI to insert the *trap* 3'UTR. The sequence encoding the *hsp86* promoter, *cytosine deaminase* and *trap* 3'UTR was released following digestion with BssHII and ligated into the backbone of *ptex150-FRT* and *exp2-FRT* to allow negative selection. *ptex150-FRT* and *exp2-FRT* constructs were linearised using NaeI, SacII and BtgZI and transfected into the NF54-FlpL line and parasites were selected on 4 nM BSD and 5-fluorocytosine (5-FC) for 14 days.

For generation of the control line: the *exp2-FRT* construct was transfected into NF45 parasites and selected on 4 nm BSD and 5-FC for 14 days.

Mosquito infection and analysis of parasite development

Seven-day old female *Anopheles stephensi* mosquitoes (originally provided by M. Jacobs-Lorena, John Hopkins University) were fed on asynchronous gametocytes diluted to 0.6% stage V gametocytemia, via water jacketed glass membrane feeders. Mosquitoes were sugar starved for 48 hours following feeding to enrich for blood fed mosquitoes. Surviving mosquitoes were provided with di-ionised water via paper/cotton wicks and sugar cubes. Oocyst numbers were obtained from midguts dissected from cold-anesthetized and ethanol killed mosquitoes 7 days post feed and stained with 0.1% mercurochrome. Salivary glands were dissected from mosquitoes (day 16-20 post bloodmeal), crushed using pestle and then glass wool filtered to obtain sporozoites that were quantified using a hemocytometer and used in subsequent assays.

Production of anti-PfLISP2 antibodies

Investigation of suitable and overlapping regions between *P. berghei* and *P. falciparum* LISP2 amino acid sequences led to the generation of an affinity purified polyclonal rabbit anti-PfLISP2 antibody targeting amino acids 889-1143 (254 amino acids), that was expressed recombinantly, purified by affinity chromatography and used to immunize rabbits (GenScript).

Immunofluorescence microscopy assay (IFA)

Asexual stages were fixed in 4% paraformaldehyde (PFA) in PBS permeabilised with 0.01% triton X-100 and probed with rat anti-HA (1:500; Roche 3F10) and rabbit anti-EXP2 (5) (1:500) in 3% normal donkey serum (NDS/ PBS). Secondary antibodies were goat anti-rabbit Alexa 594 and or anti-rat Alexa 488 (1:1000; Invitrogen). For liver stage IFAs, livers were perfused with 1x PBS and fixed in 4% (vol/vol) PFA PBS overnight before being exchanged for sucrose and snap frozen in OCT (Invitrogen). Sections of 8 mm were cut on the cryostat apparatus HMM550. Samples were permeabilised using ice cold 10% methanol:90% acetone and blocked in 3% BSA PBS. Sections were then incubated with primary antibodies; mouse anti-CSP (mAb 2A10; 1:2000), mouse anti-HSP70 (1:500), rabbit anti-PTEX150 (5) (1:500), mouse anti-EXP2 (5) (1:500), mouse anti-EXP1 (30) (mAb N1; 1:500), rabbit anti-PfLISP2 (1:500), rabbit anti-PbLSA3(17) (1:1000), rabbit anti-PbLISP2 (29) (1:500), rat anti-HA (1:500; Roche 3F10), diluted in 3% BSA PBS. Secondary antibodies were goat anti-rabbit Alexa 594 and anti-mouse or rat Alexa 488 (1:1000; Invitrogen). Samples were incubated with DAPI (4',6'- diamidino-2-phenylindole) at 1 mg/ml in PBS to visualize DNA and mounted in Prolong Gold mounting media (Invitrogen). Images were acquired on the Zeiss LSM 980 microscope.

For quantification of mean pixel intensity, Z stacks of liver stages were captured using the same exposure settings on the Zeiss LSM 980 system to allow quantitative analysis between different samples. The analysis was performed with a custom FIJI macro. Briefly, a region of interest was drawn around the parasite based on CSP staining and then membrane area and mean pixel intensity was performed on each of these regions in 488 and 594 channels for either CSP, LSA3 or PfLISP2 respectively, and statistically compared in duplicate independent experiments. For localization analyses, the periphery of the parasite was defined by a ROI based on CSP staining and an Eroded Volume Fraction (EVF) was generated creating rings of equal volume from the periphery of the parasite to the centre. The average intensity of either CSP, LSA3 or PfLISP2 in each ring and its distance from the edge of the parasite was then determined. 0 being the periphery and 1 being the centre.

Sporozoites were dissected from salivary glands on day 17 or 18 post blood meal, purified over glass wool, fixed in 4% (vol/vol) PFA 0.0075 Glutaraldehyde PBS and permeabilized in 0.01% Triton X-100 PBS. They were then incubated with primary mouse anti-CSP (2A10; 1:2000), and secondary goat anti-mouse Alexa 488 (1:1000, Invitrogen). Nuclear DNA was visualised with DAPI. Images were acquired on the Zeiss LSM 880 using mCherry excitation. Tiles were taken and the proportion of mCherry positive sporozoite was counted by eye for each knockout line compared to controls.

Immunoblotting
Synchronized late asexual schizont cultures were passed over CS magnetic columns (Miltenyi Biotech) to enrich trophozoite and or schizont-infected erythrocytes. Proteins were fractionated with saponin or equinatoxin II (31) with or without Proteinase K (see Figure S5 legend for details) and/or solubilized in Laemmli's buffer and separated through 4-12% Bis-Tris polyacrylamide gels (Invitrogen), transferred to nitrocellulose membrane and probed with primary antibodies; rat anti-HA 1:500 (Roche 3F10), rabbit anti-PTEX150 (1:500), mouse anti-EXP2 (1:1000), rabbit anti-TRAP (32) (1:1000), mouse anti-REX3 (33) (1:1000), rabbit anti-aldolase (34) (1:4000) followed by horse radish peroxidase-conjugated secondary antibodies (1:1000 (mouse) and 1:4000 (rabbit); Cell Signalling Technology) and viewed by enhanced chemiluminescence (Amersham). Western blot analysis of EXP2 expression in sporozoites: Dissected and purified NF54 sporozoites were incubated for 30 mins at either 4 °C or 37 °C in media with or without human serum. Sporozoites were centrifuged at 10,000 *g* for 55 min and both the pellet and supernatant were solubilized in sample buffer and subjected to SDS-PAGE. Uninfected salivary glands (UI SG) were dissected and incubated as above as a negative control. Lysates from Control 2 blood stages (BS) were used as a positive control for EXP2 expression. $1x10^6$ sporozoites were used per condition.

Hepatocyte culturing
HC-04 hepatocytes were maintained on Iscove's Modified Dulbecco's medium (IMDM), supplemented with 5% heat-inactivated fetal bovine serum (FBS) at 37° C in 5% CO_2. Cells were split 1:6 every 2-3 days once they reached 90% confluency.

Cell traversal assay
Cell traversal was measured using a cell wounding assay (35). HC-04 hepatocytes ($1x10^5$) were seeded onto the bottom of a 96-well plate using IMDM (Life Technologies, 11360-070). Sporozoites were added at an MOI of 0.3 and left to traverse hepatocytes for 2.5 hours in the presence of 1 mg/ml FITC-labelled dextran (10,000 MW, Sigma Aldrich). Cells were trypsinized to obtain a single cell suspension for FACS analysis. For each condition, triplicate samples of 10,000 cells were counted by flow cytometry in each of the three independent experiments.

Hepatocyte invasion assay
Assay was performed as previously described(35). Briefly, HC-04 hepatocytes ($1x10^5$) were seeded onto the bottom of a 96-well plate using IMDM (Life Technologies, 11360-070). Sporozoites were added at an MOI of 0.3 and left to invade hepatocytes for either 5 or 18 hours before being trypsinized to obtain a single cell suspension and transferred to a 96 well round bottom plate. Cells were fixed and permeabilised (BD bioscience) and stained with Alexa fluor 647 conjugated mouse anti-CSP antibody in 3% BSA permeabilizing solution. Cells were then washed in PBS and analysed by Flow cytometry. For each condition, triplicate samples of 10,000 cells were counted in each of the three independent experiments.

Humanized mice production and processing
$uPA^{+/+}$ SCID mice (University of Alberta) were housed in a virus and antigen free facility supported by the Health Sciences Laboratory Animal Service at the University of Alberta and cared for in accordance with the Canadian Council on Animal Care guidelines. All protocols involving mice were reviewed and approved by the university of Alberta Health Sciences Laboratory Animal Service Animal Welfare committee and the Walter and Eliza Hall Institute of Medical Research Animal Ethics Committee. uPA+/+-SCID mice at 5–14 days old received 10^6 human hepatocytes (cryopreserved human hepatocytes were obtained from BioreclamationIVT—Baltimore MD) by intrasplenic injection and engraftment was confirmed 8 weeks post-transplantation by analysis of serum human albumin (36, 37). For humanized mice co-infections: 3×10^5 *ptex150-FRT, exp2-FRT* and Control 1 *P. falciparum* sporozoites ($9x10^5$ total) were injected into each of 3 mice by the intravenous route. Livers were isolated 6 days post-infection from CO_2-euthanized mice and individual lobes were cut as described (38), pooled and emulsified into a single cell suspension and flash frozen in liquid nitrogen for subsequent genomic DNA (gDNA) extraction. Genomes of each parasite line was quantified using primer pairs 3 and 4. For individual humanized mouse infections: 8.0×10^5 of either *ptex150-FRT, exp2-FRT* or Control 2 *P. falciparum* sporozoites freshly dissected

from mosquito salivary glands were injected by intravenous injection into 2 humanized mice per parasite line, as previously described (36) and culled on either day 3 or 5 post infection for each parasite line. Parasite genomes were quantified using primers that would amplify the 18S gene present in each parasite line.

Measuring exoerythrocytic development in humanized mice
To quantify parasite load in the chimeric livers, gDNA was isolated from the single cell liver suspensions and Taqman probe-based qPCRs were performed as previously described (36, 38, 39). To specifically differentiate Control 1 from *ptex150-FRT* and *exp2-FRT* genomes from the same mouse samples, the oligonucleotides used are listed in Table 1. Human and mouse genomes were quantified using oligonucleotides specific for prostaglandin E receptor 2 (PTGER2) from each species, as described previously (36, 40). Sequences of primers used are provided in Supplementary Table 1. All probes were labelled 5′ with the fluorophore 6-carboxy-fluorescein (FAM) and contain a double- quencher that includes an internal ZENTM quencher and a 3′ Iowa Black® quencher from IDT. The following probes were used:
Control: 5′FAM CATACTCAGGTAC/ZEN/AGGTTAGCTCCAAAAG -3IABkFQ,
ptex150-FRT: 5′FAM GTGATAGTAATGATAAT/ZEN/GTAAATAACAATAAAGAGG-3IABkFQ
exp2-FRT: 5′FAM CTTCATTAAATTTTGTTC/ZEN/ATTAAATTTATGTTCATTAAATGTG-3IABkFQ
18S: 5’FAM TGCCAGCAG/ZEN/CCGCGGTA-3IABkFQ
hPTGER2: FAM/TGCTGCTTC/ZEN/TCATTGTCTCG/3IABkFQ,
mPTGER2: FAM/CCTGCTGCT/ZEN/TATCGTGGCTG/3IABkFQ
Standard curves were prepared by titration from a defined number of DNA copies for the amplicon for *P. falciparum ptex150-FRT, exp2-FRT*, Control, human and mouse PTGER2. PCRs were performed on a Roche LC80 using LightCycler 480 Probe Master (Roche).

Immunoaffinity chromatography and plasmepsin V-agarose
Plasmepsin V-agarose was prepared as described previously (41, 42). *P. falciparum* 3D7 parasites expressing HA-tagged plasmepsin V (PMV) from the endogenous locus were cultured to ~10% parasitemia of synchronous trophozoite stages under WR99210 selection. Cells were treated with 0.15% saponin and pellets solubilized in 1% Triton X-100 containing complete protease inhibitor cocktail. Supernatants were mixed with goat anti-HA agarose (Abcam) for 2 h and beads collected in Micro Bio-Spin columns (Bio-Rad). PMV-agarose was washed ten times with PBS containing complete protease inhibitors and stored at 4 ºC in same.

Biochemical plasmepsin V cleavage assays
Plasmepsin V (PMV) cleavage assays were performed as described previously (41-44). For fluorogenic peptide substrates, 5 µM FRET peptide (KAHRP: DABCYL-R-NK**RTLAQ**KQ-E-EDANS from Chinapeptides and DABCYL-R-GNK**ATAAQ**KQ-E-EDANS from Mimotopes; PfLISP2: DABCYL-R-RRVI**RILSGQ**HDN-E-EDANS and DABCYL-R-RRVI**AIASGQ**HDN-E-EDANS from Synpeptide; PbLISP2: DABCYL-R-RSIG**RILAE**TQHE-E-EDANS and DBACYL-RSIG**AIAAE**TQH-E-EDANS and DABCYL-R-KRPS**RLIAE**KESE-E-EDANS and DABCYL-R-KRPS**ALAAE**KESE-E-EDANS from Synpeptide) were incubated with 1 ng per well of recombinant *P. vivax* PMV produced as described (44) in digest buffer (0.01% Tween 20, 25 mM Tris HCl, 25 mM MOPS, pH 6.4). Samples were incubated at 37 °C for 4 h and were measured with an Envision plate reader (PerkinElmer) (ex, 340 nm; em, 490 nm). For CSP peptides (CYGSSSNTRVLNELNYDNA and YSLKKNSRSLGENDDGNNE from LifeTein), 2 nM peptides were suspended in digest buffer with 5 µL PfPMV-HA-agarose at 37 ºC for 8 h with shaking. Negative controls lacking PfPMV-HA-agarose (PBS alone) were included. Reactions ended when samples were passed through Micro Bio-Spin columns (Bio-Rad) to remove protease and flow through was analyzed on an Agilent 1100 modular HPLC and by LC-MS/MS using an Orbitrap LTQ mass spectrometer (see below).

Reversed phase-high pressure liquid chromatography
RP-HPLC was conducted using an Agilent 1100 modular HPLC comprised of an online degasser, piston pump, autosampler, column oven, diode array detector and fraction collector. Hewlett-Packard Chemstation software for LC/MS systems was used for instrument control and data acquisition and evaluation. Peptide samples were centrifuged at 10 000 ×*g* for 15 min at room

temperature (25 ºC) and loaded onto a C8 column (2.1mm internal diameter×100mm; Brownlee columns, Perkin-Elmer Instruments) in the presence of buffer A 0.05 % (v/v) trifluoroacetic acid (HPLC grade, Pierce) in Milli-Q water (Millipore). Buffer A was injected for 15 min to remove the breakthrough peak before elution of the bound peptides was performed in a linear gradient of 0–100% buffer B 0.05% (v/v) trifluoroacetic acid in acetonitrile (ChromAR HPLC grade, Malinckrodt Baker) over 12 min at a flowrate of 0.5 mL/min at 37 ºC.

Mass spectrometry

The flow through of synthetic peptide cleavage assays were injected and fractionated by reversed-phase liquid chromatography on a nanoflow HPLC system (1200 series, Agilent) using a nanoAcquity C18 150×0.15mm I.D. column (Waters) developed with a linear 60-min gradient with a flow rate of 0.5 μL/min at 45 ºC from 100 % solvent A (0.1 % Formic acid in Milli-Q water) to 60 % solvent B [0.1 % Formic acid, 60 % acetonitrile (Mallinckrodt Baker) 40 % Milli-Q water]. The nano HPLC was coupled online to an LTQ Orbitrap mass spectrometer equipped with a nanoelectrospray ion source (Thermo Fisher Scientific) for automated MS/MS. Up to five most intense ions per cycle were fragmented and analyzed in the linear trap, with target ions already selected for MS/MS being dynamically excluded for 3 min.

Mass spectra database searching

For peptide identification, LC-MS/MS data were searched against a custom decoy database comprising *Plasmodium* sequences from the LudwigNR nonredundant protein database (version Q211, 249 275 entries), common contaminants (239 entries) and recombinant PEXEL constructs (44 entries), as well as their reverse sequences. Mass spectra peak lists were extracted using extract-msn as part of Bioworks 3.3.1 (Thermo Fisher Scientific) linked into Mascot Daemon (Matrix Science). The parameters used to generate the peak lists for the LTQ Orbitrap were as follows: minimum mass 400; maximum mass 5000; grouping tolerance 0.01Da; intermediate scans 1; minimum group count 1; 10 peaks minimum and total ion current of 100. Peak lists for each nano-LC-MS/MS run were used to search MASCOT v2.2.04 search algorithm (Matrix Science). The search parameters consisted of variable modifications set for NH2-terminal acetylation (+42 Da) and oxidation of methionine (+16 Da). A precursor mass tolerance of 20 ppm, #13C defined as 1, fragment ion mass tolerance of ±0.7Da, and an allowance for up to two missed cleavages for nonspecific enzyme searches was used. SCAFFOLD (version Scaffold_3.6.2, Proteome Software Inc.) was used to validate MS/MS based peptide and protein identifications. Peptide identifications were accepted if they could be established at greater than 95.0% probability as specified by the Peptide Prophet algorithm (45).

Statistics

Oocysts were compared between groups using Kruskal-Wallis test with Dunn's correction whilst mosquito infection prevalence was compared using the chi-square test. Statistical analyses comparing mutants to control in co-infected mice were performed using the paired t-test and comparisons of individual mutants to control were performed using the Mann-Whitney test. Analyses were performed using Graphpad Prism 9 for macOS. $P<0.05$ was considered statistically significant.

Ethics Statement

Experimental protocols involving humanized mice were conducted in accordance with the recommendations in the National Statement on Ethical Conduct in Animal Research of the National Health and Medical Research Council and were reviewed and approved by the University of Alberta Health Sciences Animal Welfare Committee and the Walter and Eliza Hall Institute of Medical Research Animal Ethics Committee. Experimental protocols involving the HC-04 human hepatocyte cell line were reviewed and approved by the Walter and Eliza Hall Institute of Medical Research Biosafety Committee.

SI References

1. G. I. Sanchez, W. O. Rogers, S. Mellouk, S. L. Hoffman, Plasmodium falciparum: exported protein-1, a blood stage antigen, is expressed in liver stage parasites. *Exp Parasitol* **79**, 59-62 (1994).
2. A. M. Lisewski *et al.*, Supergenomic network compression and the discovery of EXP1 as a glutathione transferase inhibited by artesunate. *Cell* **158**, 916-928 (2014).
3. A. Tribensky, A. W. Graf, M. Diehl, W. Fleck, J. M. Przyborski, Trafficking of PfExp1 to the parasitophorous vacuolar membrane of Plasmodium falciparum is independent of protein folding and the PTEX translocon. *Cell Microbiol* **19** (2017).
4. P. Mesen-Ramirez *et al.*, EXP1 is critical for nutrient uptake across the parasitophorous vacuole membrane of malaria parasites. *PLoS Biol* **17**, e3000473 (2019).
5. T. F. de Koning-Ward *et al.*, A newly discovered protein export machine in malaria parasites. *Nature* **459**, 945-949 (2009).
6. J. R. Beck, V. Muralidharan, A. Oksman, D. E. Goldberg, PTEX component HSP101 mediates export of diverse malaria effectors into host erythrocytes. *Nature* **511**, 592-595 (2014).
7. B. Elsworth *et al.*, PTEX is an essential nexus for protein export in malaria parasites. *Nature* **511**, 587-591 (2014).
8. H. E. Bullen *et al.*, Biosynthesis, localization, and macromolecular arrangement of the Plasmodium falciparum translocon of exported proteins (PTEX). *J Biol Chem* **287**, 7871-7884 (2012).
9. C. M. Ho *et al.*, Malaria parasite translocon structure and mechanism of effector export. *Nature* **561**, 70-75 (2018).
10. K. Fischer *et al.*, Characterization and cloning of the gene encoding the vacuolar membrane protein EXP-2 from Plasmodium falciparum. *Mol Biochem Parasitol* **92**, 47-57 (1998).
11. P. Mesen-Ramirez *et al.*, Stable Translocation Intermediates Jam Global Protein Export in Plasmodium falciparum Parasites and Link the PTEX Component EXP2 with Translocation Activity. *PLoS Pathog* **12**, e1005618 (2016).
12. S. C. Charnaud, R. Kumarasingha, H. E. Bullen, B. S. Crabb, P. R. Gilson, Knockdown of the translocon protein EXP2 in Plasmodium falciparum reduces growth and protein export. *PLoS One* **13**, e0204785 (2018).
13. M. Garten *et al.*, EXP2 is a nutrient-permeable channel in the vacuolar membrane of Plasmodium and is essential for protein export via PTEX. *Nat Microbiol* **3**, 1090-1098 (2018).
14. T. Annoura *et al.*, Two Plasmodium 6-Cys family-related proteins have distinct and critical roles in liver-stage development. *FASEB J* **28**, 2158-2170 (2014).
15. C. F. Ockenhouse, F. W. Klotz, N. N. Tandon, G. A. Jamieson, Sequestrin, a CD36 recognition protein on *Plasmodium falciparum* malaria-infected erythrocytes identified by anti-idiotype antibodies. *Proc. Natl. Acad. Sci. USA* **88**, 3175-3179 (1991).
16. P. Daubersies *et al.*, Protection against Plasmodium falciparum malaria in chimpanzees by immunization with the conserved pre-erythrocytic liver-stage antigen 3. *Nat Med* **6**, 1258-1263 (2000).
17. M. Morita *et al.*, Immunoscreening of Plasmodium falciparum proteins expressed in a wheat germ cell-free system reveals a novel malaria vaccine candidate. *Sci Rep* **7**, 46086 (2017).
18. A. M. Vaughan *et al.*, Complete *Plasmodium falciparum* liver-stage development in liver-chimeric mice. *J Clin Invest* **122**, 3618-3628 (2012).
19. A. P. Singh *et al.*, *Plasmodium* circumsporozoite protein promotes the development of the liver stages of the parasite. *Cell* **131**, 492-504 (2007).
20. A. Coppi *et al.*, The malaria circumsporozoite protein has two functional domains, each with distinct roles as sporozoites journey from mosquito to mammalian host. *J Exp Med* **208**, 341-356 (2011).
21. Y. Orito *et al.*, Liver-specific protein 2: a Plasmodium protein exported to the hepatocyte cytoplasm and required for merozoite formation. *Mol Microbiol* **87**, 66-79 (2013).
22. M. J. Shears *et al.*, Proteomic Analysis of Plasmodium Merosomes: The Link between Liver and Blood Stages in Malaria. *J Proteome Res* **18**, 3404-3418 (2019).

23. K. S. Saliba, M. Jacobs-Lorena, Production of *Plasmodium falciparum* gametocytes in vitro. *Methods Mol Biol* **923**, 17-25 (2013).
24. M. J. Boyle *et al.*, Isolation of viable *Plasmodium falciparum* merozoites to define erythrocyte invasion events and advance vaccine and drug development. *Proc Natl Acad Sci U S A* **107**, 14378-14383 (2010).
25. M. L. Wilde *et al.*, Protein Kinase A Is Essential for Invasion of Plasmodium falciparum into Human Erythrocytes. *mBio* **10** (2019).
26. M. Ghorbal *et al.*, Genome editing in the human malaria parasite *Plasmodium falciparum* using the CRISPR-Cas9 system. *Nature biotechnology* **doi:10.1038/nbt.2925** (2014).
27. J. C. Volz *et al.*, Essential Role of the PfRh5/PfRipr/CyRPA Complex during Plasmodium falciparum Invasion of Erythrocytes. *Cell Host Microbe* **20**, 60-71 (2016).
28. T. S. Voss *et al.*, A *var* gene promoter controls allelic exclusion of virulence genes in *Plasmodium falciparum* malaria. *Nature* **439**, 1004-1008 (2006).
29. S. Itani, M. Torii, T. Ishino, D-Glucose concentration is the key factor facilitating liver stage maturation of Plasmodium. *Parasitol Int* **63**, 584-590 (2014).
30. K. R. Porter *et al.*, Immune response against the exp-1 protein of Plasmodium falciparum results in antibodies that cross-react with human T-cell lymphotropic virus type 1 proteins. *Clin Diagn Lab Immunol* **5**, 721-724 (1998).
31. K. E. Jackson *et al.*, Selective permeabilization of the host cell membrane of Plasmodium falciparum-infected red blood cells with streptolysin O and equinatoxin II. *Biochem J* **403**, 167-175 (2007).
32. T. W. Gilberger, J. K. Thompson, M. B. Reed, R. T. Good, A. F. Cowman, The cytoplasmic domain of the *Plasmodium falciparum* ligand EBA-175 is essential for invasion but not protein trafficking. *J. Cell Biol.* **162**, 317-327 (2003).
33. T. Spielmann *et al.*, A cluster of ring stage-specific genes linked to a locus implicated in cytoadherence in *Plasmodium falciparum* codes for PEXEL-negative and PEXEL-positive proteins exported into the host cell. *Mol. Biol. Cell* **17**, 3613-3624 (2006).
34. J. Baum *et al.*, A conserved molecular motor drives cell invasion and gliding motility across malaria life cycle stages and other apicomplexan parasites. *J. Biol. Chem.* **281**, 5197-5208 (2006).
35. S. Lopaticki *et al.*, Tryptophan C-mannosylation is critical for Plasmodium falciparum transmission. *Nature communications* **13**, 4400 (2022).
36. A. S. P. Yang *et al.*, Cell Traversal Activity is Required for *Plasmodium falciparum* Liver Infection in Humanized Mice. *Cell reports* **18**, 3105-3116 (2017).
37. D. F. Mercer *et al.*, Hepatitis C virus replication in mice with chimeric human livers. *Nat Med* **7**, 927-933 (2001).
38. S. Lopaticki *et al.*, Protein O-fucosylation in Plasmodium falciparum ensures efficient infection of mosquito and vertebrate hosts. *Nature communications* **8**, 561 (2017).
39. S. Y. Alcoser *et al.*, Real-time PCR-based assay to quantify the relative amount of human and mouse tissue present in tumor xenografts. *BMC Biotechnol* **11**, 124 (2011).
40. A. S. P. Yang *et al.*, AMA1 and MAEBL are important for *Plasmodium falciparum* sporozoite infection of the liver. *Cellular Microbiology* **In press** (2017).
41. I. Russo *et al.*, Plasmepsin V licenses *Plasmodium* proteins for export into the host erythrocyte. *Nature* **463**, 632-636 (2010).
42. J. A. Boddey *et al.*, An aspartyl protease directs malaria effector proteins to the host cell. *Nature* **463**, 627-631 (2010).
43. J. A. Boddey *et al.*, Role of Plasmepsin V in export of diverse protein families from the *Plasmodium falciparum* exportome. *Traffic* **5**, 532-550 (2013).
44. A. N. Hodder *et al.*, Structural basis for plasmepsin V inhibition that blocks export of malaria proteins to human erythrocytes. *Nat Struct Mol Biol* **22**, 590-596 (2015).
45. A. Keller, A. I. Nesvizhskii, E. Kolker, R. Aebersold, Empirical statistical model to estimate the accuracy of peptide identifications made by MS/MS and database search. *Analytical chemistry* **74**, 5383-5392 (2002).
